# Report on the Fifth International Conference on Natural Products for Health and Beauty (NATPRO 5) Held in Thailand, 6–8th May, 2014

**DOI:** 10.3390/nu6104115

**Published:** 2014-10-10

**Authors:** Supayang Voravuthikunchai, Peter Howe

**Affiliations:** 1 Department of Microbiology, Faculty of Science and Natural Product Research Center of Excellence, Prince of Songkla University, Hat Yai, Songkhla 90112, Thailand; 2 Clinical Nutrition Research Centre, School of Biomedical Sciences and Pharmacy, University of Newcastle, Callaghan, NSW 2308, Australia; E-Mail: peter.howe@newcastle.edu.au

## 1. Preface

The 5th International Conference on Natural Products for Health and Beauty (NATPRO 5) was held at the Moevenpick Resort and Spa Karon Beach, Phuket, Thailand on 6–8 May 2014. NATPRO was established in 2005 by Professor Maitree Suttajit, Mahasarakham University with the aim of building research networking on natural products. NATPRO 2, 3 and 4 were subsequently organized by Naresuan University, Rangsit University and Chiang Mai University in 2008, 2011 and 2012, respectively.

NATPRO 5 was organized by the Natural Product Research Center of Excellence, Prince of Songkla University under the leadership of Prof. Dr. Supayang Voravuthikunchai, and was co-hosted by University of Phayao, Thailand and the international open access journal *Nutrients*.

The conference was officially opened by the Director General, Department for Development of Thai Traditional and Alternative Medicine, Thai Ministry of Public Health and brought together some 400 participants from 30 countries with eight distinguished keynote lectures and another 20 invited lectures.

Addressing the theme of *Current Trends in Natural Products*, NATPRO 5 was an outstanding showcase of research on bioactives from plants and other natural sources, characterising their chemistry, efficacy and safety in human health and beauty applications. In a welcoming speech, Prof. Dr. Voravuthikunchai emphasised the importance of international research collaboration for the global knowledge economy, recognising that internationally mobile researchers tend to be more productive and, moreover, that a shared research interest can help to build trust. She expected NATPRO 5 to initiate partnerships with industrial sectors to enhance research capacity and innovation for social and economic growth. A significant outcome of the conference was the formation of a new regional society to promote these goals.

The following abstracts of invited and keynote lectures and selected oral and poster communications illustrate the extent of international engagement and the expertise and quality of research on natural products being undertaken in the region.

## 2. Keynote Lectures

### 2.1. Circulatory Effects of Bioactive Nutrients Deliver Cardiovascular, Metabolic and Cognitive Benefits Peter R C Howe

Numerous plant polyphenols reportedly afford multiple health benefits, ranging from anti-inflammatory to cardiovascular, metabolic and cognitive. It is becoming increasing apparent that antioxidant capacity alone cannot account for the wide variety of observed effects. An equally plausible explanation is the potential for polyphenols to enhance specific endothelium-mediated circulatory functions. Endothelial dysfunction is likely to be a primary pathogenic link between chronic conditions associated with obesity, such as diabetes, cardiovascular disease, inflammatory disorders, cognitive decline and depression. Flow mediated dilatation (FMD), a surrogate measure of endothelial function which is impaired in obesity and metabolic syndrome, is inversely related to cognitive performance. Regular consumption of marine omega-3 fatty acids, cocoa flavanols, isoflavones or resveratrol can elicit sustained improvements of FMD which may deliver not only cardiovascular but also metabolic and mental health benefits. Transcranial Doppler ultrasound can also be used to explore the link between cognitive effects of nutrients and endothelial function in cerebral arteries. This new approach is enabling us to identify nutrients with the potential to sustain mental health in an ageing population and to counteract cognitive decline in hypertension, diabetes and other chronic disorders.

### 2.2. Balancing Immunity (BIM): A New Dimension of Healthcare Pichaet Wiriyachitra

Human immune system protects the body from foreign substances, cells and tissues. An underactive or weakened immune system will expose the body to infections and diseases. An overactive immune system will lead to autoimmune diseases, inflammatory diseases and allergies. Balancing immunity (BIM) is crucial for maintaining health. T helper cells (Th cells) are a group of white blood cells that play an important role in the immune systems. In the latest understanding, balancing immunity (BIM) is believed to be brought about by balancing the activities of these Th cells, particularly, Th1, Th2, Th17 and Treg (T regulatory cells).The scientists of Asian Phytoceuticals Public Company Limited have been involved with the operation of balancing immunity (Operation BIM) by

(1) Developing formulations from the synergistic mixtures of the active ingredients from *Garcinia mangostana*, *Sesamun indicum*, *Glycine max*, *Psidium guajava* and *Centella asiatica*.
(2) Determining the effects of these formulations on Th cell activities, *in vivo*.
(3) Registering these formulations with the Food and Drug Administration as dietary supplements and providing them to consumers for balancing the body immunity and remedying ailments.

These formulations have been proven effective in solving a wide range of physical complaints. Of the most effective ones are the formulations for allergy, ulcer, infection, cyst, tumor, cancer, rheumatoid arthritis, diabetes and psoriasis. BIM by these formulations is serving as a new dimension of healthcare.

### 2.3. Challenges and Opportunities in the Globalization of African Traditional Medicines—A South African Perspective Alvaro Viljoen

African traditional medicine is one of oldest healing modalities supported by a wealth of ancient indigenous knowledge systems and modern scientific evidence. Despite a unique biodiversity and the extensive history of African traditional healing practices very few commercial entities have been developed and globalised from the African flora. From a South African perspective, the lack of research capacity in the field of natural product sciences has clearly impacted negatively on botanical product development. It is crucially important that a solid exploratory phase involving basic research should precede any commercialisation initiatives. Furthermore, developing phytomedicines based on traditional knowledge demands full compliance with local and international legislation. Several examples from the South African flora (*Harpagophytum*, Rooibos, *Pelargonium*, *Sceletium*, *Sutherlandia* *etc.*) will be discussed to illustrate the challenging plant-to-product pipeline. Each of these species has an intricate history in the commercialisation process and the importance of basic research and biosystematic studies will be highlighted as crucial steps in product development.

### 2.4. Bioprospects of Marine Microbiota—A Dynamic Source for Anti-Infectives Shunmugiah Karutha Pandian

Infectious diseases are the leading cause for morbidity and mortality in developing and under developed countries where resistance to antimicrobial agents is a common scenario. Multidrug resistance has been displayed not only by the pathogenic microbes but also by opportunistic pathogens making the infections life threatening. Antibiotics exert selection pressure over the growth of pathogens resulting in stress driven mutation and in turn development of resistance. This phenomenon forced us to look for novel strategies which not necessarily kill the pathogen but effectively control them further facilitating their clearance by the host immune machinery. In this context, the quorum sensing (QS) system—a density dependent cell to cell communication—has been targeted as an alternate strategy as it controls majority of the virulence traits in bacterial and fungal pathogens including their biofilm formation. Agents that effectively control the quorum sensing and/or biofilm formation are collectively called as anti-infectives, virulence modifying agents or anti-pathogenic agents. Epibiotic marine microbes are of recent interest as they were shown to produce novel metabolites and enzymes with unique properties. Marine microbes are believed to produce novel metabolites with bioactive potential so as to thrive the highly competitive environment. For the first time, coral ecosystem, a hitherto underexplored reserve for microorganisms, has been shown to have microbes with the ability to produce numerous antibiofilm and anti-QS agents. Similarly, studies with the epibiotic actinomycete associated with the sea weed, *Gracilaria gracilis* has been shown to produce two different metabolites active against the opportunistic pathogens *Staphylococcus epidermidis* and *Candida* sp. Apart from the epibiotic bacteria found associated with marine eukaryotes, numerous other free living bacteria isolated from diverse marine habitats like sediment soil, sea water and mangrove rhizosphere soil were shown to produce antibiofilm and anti-QS agents effective against Gram positive and Gram negative bacteria as well as against fungal pathogens of humans. Only about 1% of the marine microbiota has been discovered so far and huge wealth remains submerged in deep oceans which could be explored and exploited for the benefit of nature and mankind. Developing novel methodologies to tap the untapped microbes will unveil novel microbial species and products of human interest.

### 2.5. A New Class of Quorum Quenching Molecules from Staphylococcus species Affects Communication and Growth of Gram-Negative Bacteria Friedrich Götz

The knowledge that many pathogens rely on cell-to-cell communication mechanisms known as quorum sensing, opens a new disease control strategy: quorum quenching. Here we report on one of the rare examples where Gram-positive bacteria, the “*Staphylococcus intermedius* group” of zoonotic pathogens, excrete two compounds in millimolar concentrations that suppress the quorum sensing signaling and inhibit the growth of a broad spectrum of Gram-negative beta- and gamma-proteobacteria. These compounds were isolated from *Staphylococcus delphini*. They represent a new class of quorum quenchers with the chemical formula *N*-[2-(*1H*-indol-3-yl)ethyl]-urea and *N*-(2-phenethyl)-urea, which we named yayurea A and B, respectively. *In vitro* studies with the *N*-acyl homoserine lactone (AHL) responding receptor LuxN of *V. harveyi* indicated that both compounds caused opposite effects on phosphorylation to those caused by AHL. This explains the quorum quenching activity. Staphylococcal strains producing yayurea A and B clearly benefit from an increased competitiveness in a mixed community.

### 2.6. Process Design for Commercialization Rolf G Werner

With higher molecular weight and complexity of the molecular structure of protein therapeutics, the possibilities to improve the glycoprotein structure towards the beneficial therapeutic effect increases. Thus monoclonal antibodies provide the broadest opportunity for structural optimizations, such as selection of cell lines and clones for optimal glycosylation, ADCC, CDC activity, T1/2 and reduced antigenicity, which have to be designed prior to process development, to do it right the first time. At this stage it also has to be considered, whether bispecific antibodies to targeting two tumor receptors or tumor receptor and T-cells or tumor cells and dentritic cells, are not the better choice for an improved therapeutic effect and lower therapeutic dose. In order to allow competitive pricing all possibilities for high yield processes have to be incorporated into process development, such as efficient vector constructs, introduction of secretion elements to facilitate transport of therapeutic protein through the Golgi Apparatus, HTS media optimization and high cell density processes as well as selection of high capacity resins with high flow rates, providing overall yields of up to 90% and purities above 99.8%. All these optimizations should be done in high through put screening programs to allow testing of a brought number of parameters and to safe time. The MCB and WCB should be stable in viability, cell density and productivity over 100 passages to allow scale up and production in large scale. In down scale experiments, the DSP has to be validated for clearance of HCP, HCD, viruses, endotoxins and leachables from affinity chromatography resins. In all activities in product and process development the patient benefit and the economy of drug product should be considered for successful commercialization of the therapeutic protein in a competitive environment.

### 2.7. ^1^H NMR-Based Metabolomics and Cannabinoids Analysis of Medicinal Cannabis Trichomes during Flowering Period Nizar Happyana, Oliver Kayser*

**Figure 1:**
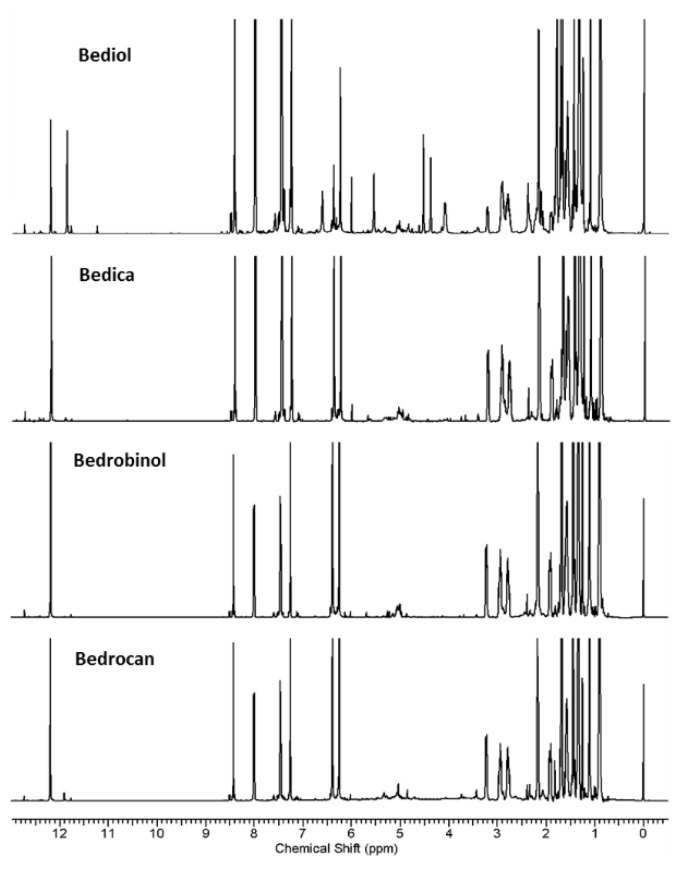
^1^H NMR spectra of the chloroform extracts of *Cannabis sativa* trichomes.

*Cannabissativa* L. trichomes are known as the main site of cannabinoids production, which are the responsible compounds for most biological activities of the plant. This study reports ^1^H NMR based-metabolomics and cannabinoids analysis of trichomes of four medicinal *Cannabis* varieties, Bediol, Bedica, Bedrobinol, and Bedrocan, in order to investigate cannabinoids production and metabolites profiles of the trichomes during the last four weeks of flowering period. Analysis of ^1^H NMR spectra (Figure 1) revealed totally six identified cannabinoids in the chloroform extracts, Δ^9^-tetrahydrocannabinolic acid [THCA], cannabidiolic acid [CBDA], cannabichromenic acid [CBCA], cannabigerolic acid [CBGA], Δ^9^-tetrahydrocannabinol [THC] and cannabidiol [CBD], and 20 compounds in the water extracts including sugars, amino acids, and other acidic compounds. Different metabolite profiles within trichomes varieties were revealed by Partial least-squares-discriminant analysis (PLSDA) models of metabolomics. Important differential metabolites in this discrimination were THCA and CBDA in the chloroform extracts, and asparagine, choline, fructose and glucose in the water extracts. Furthermore PLSDA classified trichomes of every variety based on their harvested weeks. THCA was found as an important discriminant compound in the chloroform extracts of every variety. Meanwhile, threonine, asparagine and fructose were detected as differential metabolites in the water extracts of each variety. This study indicated that *Cannabis* trichomes during flowering period produced metabolites, particularly cannabinoids in different amounts depending on time and the plant variety. Furthermore it is the first report for monitoring metabolites production in plant trichomes using ^1^H NMR-based metabolomics.

### 2.8. Anti-Infective Natural Product Discovery Using Australian Marine Invertebrates and Plants as a Bio-Diverse Resource Anthony R Carroll, Joshua Hayton, Laurence Jennings, Alan Munn, Vicky Avery

Infections are responsible for a diverse array of human diseases that can severely impact on the quality and longevity of people’s lives. Drug resistant strains of *Plasmodium falciparum*, the parasite responsible for Malaria, is a major concern in a growing number of regions around the world and is responsible for over a million deaths per year. Likewise bacteria such as *Staphylococcus aureus* and *Pseudomonas aeruginosa* are responsible for the majority of hospital acquired multidrug resistant bacterial infections and the likelihood of patients acquiring these infections while in hospital has reached alarming levels in many countries. We have specifically been screening natural products derived from Australian plants and marine invertebrates to find new drug leads and chemical probes to explore mechanisms associated with these infections. Prions are a group of infectious proteins that are responsible for a small group of fatal neurodegenerative diseases, with bovine spongiform encephalopathy (BSE) (mad cows disease) or its human form, variant Creutzfeldt-Jakob disease (CJD), most notably publicised in the media. Prions are abhorrent versions of proteins that have undergone a change in their quaternary structure which leads them to no longer being able to perform their normal biochemical function. Prion proteins can either form spontaneously, as a result of an environmental stimulus or through exposure to other prion proteins. Prions proteins are therefore considered to be infectious because they become templates for the conversion of normal proteins into their mis-folded, infective form. Prion proteins are resistant to enzymatic degradation which results in them exponentially building up in tissues where they form amyloid plaques. There is mounting evidence that prion proteins may also be linked to other diseases such as cancers, and various neurological diseases, suggesting a potential infectious basis for these diseases. Studies using yeast prions have demonstrated that small molecules can be effective at curing prion infections. We have recently started a project to find natural products from marine invertebrates to use as tools to study prion infections and the mechanisms associated with their curing action. This presentation will focus on some of our recent discoveries and highlight specific screening, isolation and structure determination methods used in our antiprion research. This presentation will focus on some of our recent discoveries and highlight specific antimalarial, antibacterial and antiprion studies.

## 3. Other Invited Lectures

### 3.1. Synthesis and Biological Evaluation of Natural Product Analogues and Hydrogenated Benzo[j]phenanthridine-7,12-diones as Anti-Tuberculosis Agents Pieter Claes, Davie Cappoen, Roel Antonissen, Vanessa Mathys, Luc Verschaeve, Kris Huygen, Norbert De Kimpe

Pentalongin, a 3,4-dehydropyranonaphthoquinone isolated as the active principle from the roots of the Central East African medicinal plant *Pentas longiflora*, formed the basis of a study on structural modifications of heteroatom-analogues of the 2-azaanthraquinone skeleton in order to investigate routes toward new antituberculous agents. This study comprised isolation of natural products, synthetic work on a defined library of natural product analogues and biotesting. A series of new *N*-analogues of pentalongin, 2-azaanthraquinones, benz[g]isoquinoiline-3,5,10(2*H*)-triones, tetracyclic naphtho[3,2,1-*de*]isoquinoline-4,7-diones, benzo[*h*]pyrido[3,4,5-*kl*]-1,2,3,4-tetrahydroacridine-5,8-diones, tetrahydro- and octahydro-benzo[j]phenanthridinediones were synthesized. *In vitro* testing of these compounds for their activity against *Mycobacterium tuberculosis* and against clinically relevant mycobacterium species, e.g., *M. bovis*, *M. avium* and *M. ulcerans*, at submicromolar concentrations showed promising results which will be unravelled. Especially octahydro-benzo[j]phenanthridinediones gave promising results on the antimycobacterium activity. The results of toxicological studies of these bioactive compounds will be disclosed as well. The susceptibility of a multi-drugresistant strain toward octahydro-benzo[j]phenanthridinediones and the ability of the latter compounds to target intracellular replicating M.tb was observed. These data led to the conclusion that highly potent antimycobacterial 1,2,3,4,8,9,10,11-octahydrobenzo[j]phenanthridine-7,12-diones as new leads against *Mycobacterium tuberculosis* were made accessible.

### 3.2. Development of Bioactive Substances from Plants Based on Enzyme Inhibition Ki Hun Park

Enzymes are biological molecules that play key roles in most metabolitic process that sustain life. Many drugs and nutraceutics are enzyme inhibitor because inhibition of an enzyme activity can involve to correct a metabolic imbalance and to protect disease. This presentation demonstrates the development of inhibitors against neuraminidase, acetylcholinesterase, α-glucosidase and xanthinn oxidase from various plants. The first is about that *Flemingia philippinensis* showed potent inhibitory activity toward bacterial neuraminidase that is one of the key enzymes involved in pathogenesis of inflammation during infection. The prenylated isoflavones were proved to be responsible for neuraminidase inhibition of target plant. Secondary, *Paulownia tomentosa* fruits were found to display significant inhibition against cholinesterase that are strongly linked with Alzheimer’s Disease. The geranylated flavanones were proved to be active component, and showed mixed inhibition kinetics as well as time-dependent. The binding affinities of these compounds to hAChE were investigated by monitoring quenching of inherent enzyme fluorescence. The affinity constants (*K*_SA_) increased in proportion to inhibitory potencies. Thirdly, an ethanol extract of the seedcase of mangosteen, whose most abundant chemical species are xanthones, showed potent α-glucosidase inhibitory activity (IC_50_ = 3.2 μg/mL). A series of isolated xanthones demonstrated modest to high inhibition of α-glucosidase with IC_50_ values of 1.5–63.5 μM. The actual pharmacological potential of the ethanol extract was demonstrated by showing that it could elicit reduction of postprandial blood glucose levels. Finally, the chalcones in *Angelica keiskei* were found to be responsible for the xanthin oxidase inhibition shown by this plant. The most potent inhibitor, xanthoangelol inhibited xanthin oxidase with an IC_50_ of 8.5 μM. Chalcones exhibited mixed-type inhibition characteristics. The specific quantification of chalcones was accomplished by multiple reaction monitoring (MRM) using a quadruple tandem mass spectrometer.

### 3.3. Isoflavone C- and O-Glucosidase Metabolism by Human Intestinal Bacteria Mihyang Kim, Jaehong Han*

Human intestinal microbiota is called an additional organ because its metabolic capacity is considered equal to that of liver. However, the metabolism by intestinal microbiota is depending on individuals due to different host microbial symbiotic states. To establish the link between *in vitro* biological activity and pharmacokinetics of flavonoids is of great interest and underdeveloped research area. We have isolated three new bacteria, MRG-IFC-1, MRG-IFC-2 and MRG-IFC-3, that catalyzed the hydrolysis of *C*-glycosidic bond of puerarin, daidzein-8-*C*-glucoside, from human fecal samples. They were identified as *Enterococcus* and *Lactobacillus lactis* species, based on 16S rDNA sequence analysis. The isolated bacteria hydrolyzed isoflavone *C*- and *O*-glycosides, as well as apigetrin, flavone *O*-glycoside.

### 3.4. The Health Benefits of the Boiled Water Extract of Dried Flowers of Aegle marmelos; a Traditional Beverage Thusharie Sugandhika Suresh

*Aegle marmelos* Correa (L.) is a highly reputed medicinal plant in the traditional systems of medicine of Asian countries. Although many parts of this plant have been extensively investigated, the flower has gained little attention. The boiled water extract of the dried flowers of *Aegle marmelos* is a popular traditional beverage in Sri Lankan rural areas used in place of black tea and possesses soothing and calming effects. During the recent past, it has become popular among the urban community. The hypoglycaemic, anti-oxidant, anti-inflammatory and organoleptic properties of this traditional drink were evaluated in Wistar rats, healthy volunteers and diabetic patients. In diabetic patients, after consuming the test extract for 14 days, the fasting serum glucose concentration was reduced by 20.0% (*p* = 0.001) and the post glucose load serum glucose concentation was reduced by 35.5% (*p* = 0.001). Both fasting serum insulin levels [(*p* < 0.05) by 27.5%] and post glucose load insulin levels [(*p* = 0.001) by 62%] were increased. No adverse effects were observed after the experiment and the serum levels of tested enzymes, creatinine and Hb were not significantly altered in humans.The anti-oxidant activity was tested in rats, by TBARS assay and following 4 weeks of adminstration of the herbal drink, the percentage reduction of serum TBARS of Test group was 36.02% ± 5.8% (*p* = 0.0001) in comparison to control. The anti-inflammatory activity when studied in rats proved to be exerted by inhibition of production of nitric oxide, anti-histamine effect and membrane stabilization activity. Overall this traditional beverage can be recommended as a functional food with medicinal values. Acknowledgements: The University of Sri Jayewardenepura is acknowledged for financial assistance.

### 3.5. Effect of the Extract from Phellinus igniarius on Acrolein Toxicity in Vitro and on Protection in Stroke Model Mouse Papawee Suabjakyong, Ryotaro Saiki, Leo J. L. D. Van Griensven, Frankie Chan, Kazuei Igarashi, Toshihiko Toida*

*Phellinus igniarius* is a mushroom in the family of *Hymenochaetaceae basidiomycetes*, has been used as traditional medicine in China, Japan and Korea and other Asian countries for many years. Over the past several decades, researchers have reported that the extracts show anti-inflammatory, anti-oxidative activities and also have the ability to be an anti-tumor. Furthermore, current knowledge about effect of crude extract from *Phellinus igniarius* on stroke is still limited. Stroke is neurodegenerative disorder in which oxidative stress is a key hallmark. The oxidative stress might be one of pathogeneses at early stage in the disease, and can exacerbate its progression. On the contrary, there is a very important report published by Kazuei Igarashi’s group that acrolein produced from polyamines *in vivo* is a major factor responsible for cell damage by its oxidative stress. Based on this fact, we have investigated that the effect of the extracts from *Phellinus igniarius* for treatment/protection of stroke. The toxicity of acrolein was compared with that of reactive oxygen species using a mouse mammary carcinoma (FM3A) cell lines. Complete inhibition of cell growth was accomplished by 10 µM acrolein and 200 µM H_2_O_2_ suggesting that FM3A cell lines were prevented by ethanol extract of *Phellinus Igniarius* from Amazing Grace Health Products Limited Partnership (Bangkok, Thailand) at 0.5 µg/mL. Polyphenol extracts of *Phellinus* *igniarius* were used for prevention of acrolein toxicity in mouse neuroblastoma (Neuro2a) cell line. The results have suggested Neuro2a cells were prevented from acrolein toxicity at 2 and 5 µM by polyphenol extract at 0.5 and 2 µg/mL. Furthermore, mice were induced experimental stroke by photoinduction after injection of Rose Bengal followed by laser irradiation, and treated with intraperitoneal 20 µg/kg polyphenol extract from *Phellinus igniarius* that the extract could reduce infarction volume to 37.8% compared with untreated mice as 100%. These observations may strongly suggest that polyphenol extract of *Phellinus igniarius* affords to serve as a substance for new neuroprotective treatment of ischemic stroke. The investigation for neuroprotective mechanisms of the extract of *Phellinus igniarius* is currently in progress.

### 3.6. Medicinal Plants in Current Therapeutics: Are We Missing the Last Chance? Christophe Wiart

This work provides a critique of a dominant patternin current medicinal plant uses in therapeutic—which is to give first place to synthetic molecules. It argues that suggestions that we have moved away from medicinal plant understandings and failed to use medicinal plants for the wellbeing of humanity. This work paper demonstrate that medicinal plants have throughout history assisted mankind against diseases till today where there use and controls have been shifted from herbalist, to pharmacist and currently industrials This way of separating man from his knowledge in plants underlies new global configurations. In other words globalization tends to attenuate medicinal plant knowledge in order to monopolize health care systems. The article concludes by making some comments on this trend, and suggests that if nothing is being done to train professional and medicinal plants and to ensure the teaching of medicinal plants in Universities, the chance to win the battle against diseases and to form skilled pharmacists and herbalists shall be missed.

### 3.7. New Therapeutic Compounds from Natural Products by Using Cell Signal Transduction for Malignant Pediatric Tumors Takashi Suzuki

The result of treatment for malignant pediatric tumors including leukemia is improving by conventional multimodal treatment with strong chemotherapy, surgical resection, radiotherapy, and bone marrow plantation. However, the patients of advanced neuroblastoma, metastatic Ewing’s sarcoma family of tumor (ESFT), and osteosarcoma have an extremely poor prognosis till now. Therefore, novel therapeutic strategies are urgently needed to improve the prognoses of these patients. Apoptotic cell death is a key mechanism for normal cellular homeostasis. Intact apoptotic mechanisms are pivotal for embryonic development, tissue remodeling, immune regulation, and tumor regression. Genetic aberrations disrupting programmed cell death often underpin tumorigenesis and drug resistance. Moreover, it has been suggested previously that apoptosis or cell differentiation proceeds to spontaneous regression in early stage neuroblastoma. Therefore, apoptosis or cell differentiation is a critical event that expects the prognoses of patients. We extracted many compounds from some natural plants (*Hyptis incana*, *Lycaria puchury-major*, *et al.*) or synthesized indirubin derivatives, vitamin K3 derivatives, and GANT61, and examined the effects of apoptosis, cell differentiation, and cell cycle by using these compounds for neuroblastoma and ESFT cell lines. Some compounds were very effective for these tumor cells. Our results suggest that they may be applicable as an efficacious and safe drug for the treatment of pediatric tumors.

### 3.8. Polyphenolsdecreased DNA Methyltransferase Expression in Hydroxyl Radical Treated Murine Adipocytes Yeong Rhee*, Sherri Stastny

DNA methylation is one of the major epigenetic control mechanisms of gene expression affecting physiological functions. Reactive oxygen species (ROS) cause oxidative damage to DNA or cells affecting physiological functions. Polyphenols have health benefits attributing to anti-oxidant activity, and flaxseed (lignan: secoisolariciresinoldiglucoside, SDG) and tea (epigallocatechin-3-gallate, EGCG) are good sources of polyphenols. It was hypothesized that ROS will increase oxidative damages leading to increased DNA methyltransferase (DNMT) gene expression. Thus, anti-oxidant, such as flaxseed lignan, treatment will lower ROS induced oxidative damages and lead to decreased DNMT expression. *In vitro* DNMT gene expression was measured using murine adipocytes. Adipocytes (1 × 10^7^) were treated withSDG, EGCG, orcatalase (control anti-oxidant), and then treated with hydroxyl radical. Afterhydroxyl radical treatment, total RNA was extracted and purified using an RNeasy Mini kit. DNMT1, DNMT3a, and DNMT3b gene expression by real-time PCR was measured using β-actin as the endogenous control. The fold change in expression of the DNMTs was calculated using the 2^−ΔΔCT^ method. DNMT1, DNMT3a, and DNMT3b expression was significantly decreased in adipocytes treated with SDG, EGCG, or catalase compared to control adipocytes. Although there were no significant differences among SDG, EGCG, and catalase treatments, SDG 50 µM or SDG 100 µM showed the greatest decrease in DNMTs expression.Data demonstrated that polyphenols decrease DNMTs expression in hydroxyl radical treated adipocytes. How DNMTs expression was decreased by polyphenols, especially flaxseed lignan, is unknown and needs further study.

### 3.9. Biofango, Modified for Original Style from Italian Fango, a Method to Use Hot Spring Water for Health Promotion Kenji Sugimori*, Maiko Okajima, Mizuno Oowada, Shiv Shankar

Fangotherapy is one of the medical treatments used under the medical doctor’s supervisor of a hot spring in Europe. This treatment is using peloids maturated with natural hot spring water. In Japan, hot springs are almost always used for “taking a bath” only. Our research focused on Fango found in Abano Italy, and modified into “Japanese style Fango” made by hot spring water with maturated peloids. Hyperthermia and some medical effects were checked under treatment with maturated peloids, and the effects were compared with hot spring water only and with boiled tap water. Fango is the best treatment for keeping normal responses of blood flow and blood pressure at the thigh, and for keeping good thermal effects on the body, especially for the back of the body after 50 min. Two kinds of Fango were made using either hot tap water or hot spring water. After treatment of Fangotherapy using the double-blind method, a medical questionnaire was provided for each test subject. The results of the questionnaire show that the hot spring Fango is more effective than hot tap water Fango. According to these results, Fango is the best treatment for a body-friendly by hot spring water. Thermophilic microorganisms grew during the maturation with hot spring water, and the extract, especially for glycolipids, is an important factor for reducing inflammation on Fango. We also have evidence, *in situ*, of the glycolipids remaining between particles of peloids. The original Japanese Fango has been named Biofango^R^.

### 3.10. Determination of d-saccharic Acid-1,4-lactone (DSL) in Fermentation Tea (Kombucha) by Capillary Electrophoresis Surapol Natakankitkul*, Panee Sirisa-ard, Sakunnee Bovonsombut, Chatchai Kitipornchai, Suwalee Kiatkarun

Kombucha or fermentation tea beverage produced from symbiosis of yeast species and acetic acid bacteria, is a popularly health tonic around the world today. d-saccharic acid-1,4-lactone (DSL), a component of kombucha, inhibits the activity of glucuronidase, known as an enzyme indirectly related with liver cancer. In this research, the tea fermentation was traditionally carried out by inoculating a previously grown culture into a freshly prepared tea decoction and incubated statically under aerobic conditions using 1.0% local tea from Northern Thailand, 10.0% sugarcane and 10.0% fungus broth at room temperature for 7–40 days, then filtered and pasteurization. After subsequent fermentation process procedure above, sampling was performed periodically in each jar. We founded various yeasts, acetic acid bacteria and lactic acid bacteria in floating cellulosic pellicle layer and the liquid broth after 7 days of fermentation. The ethanol content was determined by gas chromatography (GC) and Ebulliometer^®^ was founded between 0.5%–5.0% v/v. For safety of consumers, all samples were checked toxic substance such as methanol by using GC-MS. The total antioxidant of kombucha samples capacity was measured in terms of free radical–scavenging activity by the 1-diphenyl-2-picrylhydrazyl (DPPH) Radical decolorization method were between 0.221–0.512 mg gallic acid/mL and the total phenolic content was determined by the Folin-Ciocalteau method were between 0.291–0.854 mg gallic acid/mL. Using a capillary electrophoresis (CE) for the separation and determination of DSL in kombucha samples was carried out on CE System: PA 800 *plus* Pharmaceutical Analysis System (Beckman Coulter Inc., Brea, CA, USA) with optimized conditions of 50 cm effective length capillary at a separating voltage of 30 kV in 40 mmol/L borax buffer (pH 6.5) containing 30 mmol/L SDS and 15% v/v methanol. The relationship between peak are and concentration of DSL was determined by UV absorption at wavelength 190 nm with the linear range of 25–200 µg/mL and a detection limit of 25 µg/mL. The electropherogram or fingerprint of the different fermented type’s tea such as; green tea, oolong tea and black tea were established. We have successfully applied a simple CE method for quantitative evaluation of polyphenol or catechins and DSL in various fermented conditions and different kombucha products.

### 3.11. The Developmental Integration between Phytotherapy and Oriental Medicine Koichiro Tanaka

When we learn a traditional medicine, such as Oriental medicine, it is necessary to understand not only technical aspect of it, but also its theory about human body, life and the universe. Here, we compared the usage of herbal plants between Phytotherapy which was “modernized” traditional medicine in German and Oriental Medicine, especially Kampo (Traditional Japanese Medicine) and Traditional Chinese Medicine (TCM) which were practiced mainly in East Asia and showed that even same or related species of plants could be used differently because both have different theories. To understand more than one traditional medicine will help us understand various aspects of herbal plants. Moreover, theories of traditional medicines and scientifically researches are indispensable to achieve the new integration among traditional and modern medicines.

### 3.12. Natural Products from Psychoactive Plants as New Drug Leads for CNS and GI Disorders Jordan K. Zjawiony

Psychoactive natural products play an important role in the discovery and development of new drugs for the treatment of central nervous system (CNS) disorders. They have led to discovery of number of new receptors systems and their endogenous ligands. Development of useful biological probes of CNS receptors helped in understanding of causes of many CNS disorders. For many of them we do not know yet the mechanism of action in CNS, and their toxicity to vital organs and systems of human body. Our research is focusing on identification of plant metabolites responsible for CNS activity and designing new ligands with high affinity to CNS receptors. Studies on psychoactive plants of abuse such as *Salvia divinorum*, *Calea zacatechichi*, *Lagochilus inebrians* and *Heimia* *salicifolia* made possible for identification of several secondary metabolites potentially responsible for CNS activity of these plants. Major effort of our research is on chemical modification of salvinorin A, the major active metabolite of *Salvia divinorum*. Salvinorin A is an agonist with high affinity to kappa opioid receptor (KOR). Molecular modeling of salvinorin A binding site led us to design and synthesis of 22-thiocyanatosalvinorin A, the most potent ever known KOR agonist, which covalently binds to the receptor. This finding helped to better understand the binding site and further development of potential CNS drugs with salvinorin A scaffold. In the course of our studies we have found that the simple chemical modification of the structure of salvinorin A significantly alters the pharmacological profile from CNS to gastrointestinal (GI) activity.

### 3.13. Anti-Proliferationand Acute Toxicity Studies of Curcuma manga Rhizomes Lee Guan Serm*, Hong SokLai, Hong Gin Wah, NurfinaAznam, HashimYaacob, Mahmood Ameen Abdulla Hassan, Norhaniza Aminudin, Sri Nurestri Abd Malek

Anti-proliferation studywas conducted on the crude methanolic and fractionated extracts (hexane, ethyl acetate and water) of *Curcuma manga* rhizomes (Zingiberaceae). Sulforhodamine B (SRB) cytotoxicity assay was used to evaluate the cell toxicity on the crude methanolic extracts and its fractions against two human colon carcinoma cell lines, HCT116 and HT29. Chemical and *in vivo* acute toxicity investigations were then directed to the cytotoxic active fractions. The crude methanolic and fractionated extracts (hexane and ethyl acetate) were found to exert good inhibitory effect against the HCT116 and HT29 cells with IC_50_ values less than 20.0 μg/mL. Four pure compounds, namely (*E*)-labda-8(17),12-diene-15,16-dial, (*E*)-15,16-bisnorlabda-8(17),11-diene-13-one, coronarin-D methyl ether and β-sitosterol were isolated from the hexane fraction whilst the ethyl acetate fraction afforded six compounds, namely (*E*)-labda-8(17),12-diene-15,16-dial, (*E*)-15,16-bisnorlabda-8(17),11-diene-13-one, zerumin A, curcumin, demethoxycurcumin and bis-demethoxycurcumin. *In vivo* acute toxicity study on the methanolic extract and hexane fraction was conducted on adult male and female Sprague Dawley rats and was found to have no acute toxicity. No abnormalities were detected in the serum of treated rats compared to the control rats following low and high dosages treatments.Selected pure compounds were further evaluated for their anti-proliferation activity against HCT116 and HT29 cells via SRB cytotoxicity assay. The mode of cell death of HCT116 and HT29 cells caused by the compounds will be investigated via cell morphological study, Annexin V-PI staining assay and cell cycle analysis. The findings of the present study support the common belief that ethnopharmacological selection of rhizomes *C. manga* is useful for the treatment of cancer.

### 3.14. Known Unknown of Natural Products toward Rescue from NSAID-Associated GI Damages; Polyunsaturated Fatty Acid and S-Allyl Cysteine Ki Baik Hahm*, Napapan Kangwan, Jing X Kang

*n*-3 Polyunsaturated fatty acids (*n*-3 PUFA) had been applied in diverse clinical conditions including inflammatory diseases, atherosclerosis, degenerative diseases, anti-aging as well as cancer preventive purpose supported with its anti-inflammatory and anti-oxidative actions. Although increasing levels of arachidonates and prostaglandins through COX-2 had been responsible for GI injury associated with non-steroidal anti-inflammatory drugs (NSAIDs), there is no clear result whether the n3-PUFAs rich in docosahexanoic acid (DHA) and eicosapentanoic acid (EPA) can be solving strategy. Using *fat-1* transgenic mice, which can synthesize n3-PUFA owing to over-expression of 3-desaturase, we investigated whether *n*3-PUFA can attenuate NSAID-induced GI injury. Wild-type C57BL/6 and *fat-1* transgenic mice were deprived of food 24 h before indomethacin and treated with 20 mg/kg indomethacin or vehicle by gavage and killed after 16 h for gastric injury and with 10 mg/kg indomethacin of 2 days for duodenal injury, respectively (*n* = 12). Indomethacin induced erosive and ulcerative lesions in GI tract of wild-type C57BL/6 mice, whereas gross and pathologic scores were significantly decreased in *fat-1* transgenic mice. The expressions of inflammatory genes, adhesion molecules, apoptotic executors were significantly lower in the *fat*-*1* mice compared to wild-type littermates. To correlate between n-3 PUFAs levels and protection from NSAIDs injury, the levels of each fatty acid were measured by gas chromatography and there was significant correlation between mucosal injury and *n*-3 PUFAs levels (*p* < 0.001). All of our result provided the rationale to develop either *n*-3 PUFAs combined-NSAIDs or co-administration regimen to secure the safety from NSAIDs-induced GI injury. Molecular pathogenesis and pharmacology how *n*3-PUFA showed protection will be shown. In another trial, we have established rescuing from NSAID-induced GI damages with synthetic S-allyl cysteine (SAC) and other natural products. All of these efforts, all known unknowns, may open healthy future to secure NSAID-induced GI damages as well as the development of safer NSAIDs.

### 3.15. Quorum Sensing Inhibitory Potential of Syzygium jambos (L.) Alston and Syzygium antisepticum (Blume) Merr. & Perry against Gram-Negative Bacteria Khadar Syed Musthafa*, Jongkon Saising, Supayang Piyawan Voravuthikunchai

Chemical signal mediated quorum sensing (QS) plays a vital role in the regulation of phenotypic characters expression in bacteria. In Gram negative bacteria, a chemical signal known as acyl homoserine lactone (AHL) dependent QS regulates expression of many factors, most importantly the production of virulence factors. These virulence factors facilitate the bacterial pathogens to establish successful infections to host. Consequently, interfering with AHL activity could lead to reduction in bacterial virulence and might be an alternative approach to control emerging bacterial infections without leading to any resistance development. Therefore, in this present study, it was attempted to evaluate the quorum sensing inhibitory (QSI) potential of *Syzygium jambos* (L.) Alston and *Syzygium antisepticum* (Blume) Merr. & Perry on AHL dependent QS in bacteria. *Chromobacterium violaceum* DMST 21761 and *Pseudomonas aeruginosa* ATCC 27853 were used as target bacterial strains. The ethanolic extract of *Syzygium jambos* and *Syzygium antisepticum* leaves at 500 µg/mL concentration caused a visible inhibition in AHL dependent violacein pigment production by *C. violaceum*. The extracts at the same concentration reduced AHL dependent pyoverdin production in *P. aeruginosa*. Swimming and twitching motilities of *P. aeruginosa* were also reduced when treated with these extracts. Further, *Syzygium jambos* extract showed 32% reduction in staphylolytic activity. Thus, the results revealed that ethanolic extract of *Syzygium jambos* and *Syzygium antisepticum* leaves could inhibit AHL dependent QS of bacterial pathogens. The extracts involving QSI potential will be further explored for their phytochemical constituents which may lead to the identification of anti-pathogenic drug to control emerging bacterial infections.

### 3.16. Cardio- and Neuro-Protective Functions of Bioactive Flavonoids Present Cool Climate Fruits H.P. Vasantha Rupasinghe

Flavonoids are naturally occurring plant secondary metabolites found in many plant parts specially fruits such as apples. Evidence exists that dietary flavonoids have increased the anti-oxidant capacity in the plasma and tissue of rats, birds, pigs, and humans. In apple, flavonoids such as quercetin glycosides, epicatechins and chalcones are the most predominant. Besides many recent advancements in understanding the flavonoid metabolism, mode of action of these flavonoids by gene expression and modulation of cell signal transduction yet to be discovered. A novel mechanism for flavonoid-induced cytoprotection by means of elevated lipid synthesis and inhibition of membrane lipid oxidation that may enhance plasma membrane integrity and promote membrane repair after oxidative injury has been investigated. Recently, we have also demonstrated physiological functions of apple flavonoids in relation to cardiovascular and brain health. Apple flavonoids showed lipid lowering and anti-inflammatory properties in experimental animals of hamsters and Wistar rats. Apple flavonoids inhibited angiotensin converting enzyme, which is a key enzyme that produce angiotensinogen II, a known vasoconstriction factor associated with hypertension. When the apple flavonoids was supplemented in the diets of lipopolysaccharides (LPS)-induced inflammation possessing hyperlipidemic Whister rates, hepatic and plasma levels of pro-inflammatory cytokines were reduced. Oral administration of apple flavonoids once daily for at least three days prior to hypoxia ischemia markedly reduced subsequent motor impairments, brain damage and inflammation after an experimental stroke. Overall, apple flavonoids have exhibited strong biological functions that have potential for the prevention of cardiovascular and neurodegenerative disorders.

### 3.17. Concentrated Emulsion Formulation of Tobacco Extract for Use as Pesticide Thanaporn Amnuaikit*, Chalermkiat Songkram, Luelak Lomlim, Jindaporn Puripattanavong

It has been known that tobacco extract is derived from tobacco leaves (*Nicotiana tabacum* Linn., Solanaceae) and used as ecological or green pesticide with environmental friendly. However, tobacco extract contains ingredients which could easily volatize and has unappealing appearance (brown syrupy mass and strong odors). This study intends to develop the concentrated emulsion formulation which shows good physicochemical properties and efficacy of product in order to scale up for commercial purpose. Niocotine was used as an active ingredient marker in a suitable condition of high performance liquid chromatography (HPLC) system for analyzing chemical determination and stability studies. The tobacco extract was stable under acid base and heat condition. The concentrated emulsion of tobacco extract composed of 10% w/w of nicotine was made by simple mixing with fixed oil (palm oil) and emulsifier (Tween and Span). Under condition of temperature 25 ± 1 °C and 70% RH for 6 months, the percent amount of nicotine in the product still remained in acceptable level including physically stable appearance of product. The efficacy of product when the dilution is 100 time of its product could kill aphid without burned of the plants trails in the agriculture field. Therefore, this formulation has a potential for commercial product as pesticide.

### 3.18. Forskolin and Isoforskolin from Native Plant Coleus forskohlii Play Multiple Biological Roles Fukai Bao

Forskolin is labdane diterpene produced by the Indian plant *Coleus forskohlii*. Forskolin is commonly used to raise levels of cyclic AMP (cAMP). It resensitizes cell receptors by activating the enzyme adenylyl cyclase and increasing the intracellular levels of cAMP. It is an important signal carrier necessary for the proper biological response of cells to hormones and other extracellular signals. It is required for cell communication in the hypothalamus/pituitary gland axis and for the feedback control of hormones. cAMP acts by activating cAMP-sensitive pathways such as protein kinase A and Epac. Forskolin is a vasodilator which may help to decrease blood pressure. Applied with rolipram, forskolin provides a route to inhibit colon cancer cell growth and survival. These two drugs also work together to induce long-term potentiation in neuronal populations. There have been clinical studies examining the effectiveness of forskolin as a weight loss aid. The study also observed forskolin’s role in significantly increasing lean mass, bone mass, and testosterone in the overweight and obese men involved. This research has led to companies marketing forskolin as a body building supplement. Forskolin may be helpful in controlling the underlying cause of glaucoma. Successful use of forskolin to reduce intraocular pressure may be due to its unique ability to stimulate adenylate cyclase activity and increase cAMP which regulates and activates critical enzymes required for the cellular energy required to move fluid out of the eye. Isoforskolin was isolated from *C. forskohlii* native to Yunnan in China. It is identified as one analog of diterpene forskolin (FSK) which comes from the Indian plant *C. forskohlii*. Phytochemists found that the Yunnan native plant *C. forskohlii* contained rich ISOF but not FSK, so our interest has focused on the bioactivity of ISOF. Recently, ISOF was reported to activate adenylyl cyclase (AC) isoforms 1, 2 and 5. Yang’s study demonstrated that ISOF increased cAMP level in rat liver homogenate, and relaxed the histamine induced contraction of isolated guinea pig lung and trachea smooth muscle. Isoforskolin pretreatment attenuates acute lung injury induced by lipopolysaccharide (endotoxin). In human mononuclear leukocyte, isoforskolin and dexamethasone pre-incubation lowered lipopolysaccharide (2 µg/mL) induced secretion of the cytokine TNF-alpha, and interleukins (IL)-1 beta, IL-6, and IL-8. In conclusion, pretreatment with isoforskolin attenuates lipopolysaccharide-induced acute lung injury in several models, and it is involved in down-regulation of inflammatory responses and proinflammatory cytokines TNF-alpha, IL-1 beta, IL-6, and IL-8. Our results showed that ISOF can inhibit proinflammatory cytokine production of murine macrophages activated by LPS and Lyme disease spirochete outer membrane potein BmpA.

### 3.19. Healing of Skin Aging, a Correlation with Isoflavon Joshita Djajadisastra

The most significant changes associated with skin aging occur in the dermis. Although the epidermis is thin and becomes less hydrated with age, aspects related to keratinization are not altered. Keratin filaments, membrane-coating granules, and keratohyaline granules, involved in both the epidermis keratinization process and epidermis stabilization, are present in normal amounts. Aging also produces changes in a range of cells and compounds. Fibroblasts, melanocytes, and langerhans cells decrease in density, number, and/or activity. To significantly reduce or prevent changes observed with aging, the primary target of action should be the dermis-epidermis junction and the dermis tissues. If the active compound is a collagenase inhibitor, it needs to penetrate the cell membrane. On the other hand, if the intention is to influence collagenase gene expression, the active may need to penetrate the nucleus. The challenge in this approach is to maintain a sufficient concentration of the active at the epidermis-dermis junction. Once this junction has been reached, an active compound can be readily absorbed into the blood circulation from surrounding blood vessels. Genistein is a kind of Isoflavone, isolated as genistin (glycosidic-bound to sugar) from Fruits of Sophora japonica, and after an enzymatic process it becomes an aglycone (without sugar). It works as an Anti-wrinkle agent in cosmetics, as well as works for the cure of UV damages and inhibition of 5-α reductase. In most plants, Genistein exists as a glycosidic (bound to sugar) type, such as Genistin or Sophoricoside. But aglycone (without sugar) type is much more active and permeable to skin than glycosidic type. The ability of permeation should be evaluated by Franz Difussion Cell, considering that the active compound have specific physico-chemical properties.

### 3.20. Health Benefits of Omega-3 Fatty Acids from Plants as Compared to Fish Oil: A Research Review Maitree Suttajit

Omega-3 fatty acids are essential fatty acids which are important for metabolismand health benefits. Humans and animals cannot synthesize long chain omega fatty acids such as α-linolenic acid (ALA), eicopentaenoic acid (EPA) and docosahexaenoic acid (DHA). ALA has various metabolic functions and preventive effects of cardiovascular disease, inflammation and other chronic diseases. Our body can enzymatically convert ALA to EPA and DHA respectively. ALA is mostly found in vegetables, plant seed oils and nuts. Especially perilla seed (PS) oil contains mostly ALA (55%–65%). DHA and EPA are commonly found in marine and fish oils. Deep sea fish and marine animals are accepted to be the main source of ALA, EPA and DHA. However, fish oil may be contaminated with methy mercury and other toxins, while several plant seeds are rich of ALA rather than linoleic acid (LA, ω-6 fatty acids). The consumption of more saturated fatty acids and LA from animal meats but less ALA may lead to a risk of several chronic diseases, Since vegetarians and vegans who eat no meats and fish receive ALA and polyunsaturated fatty acids only from plants, we wonder whether fatty acids from plants instead from fish could maintain or affect their normal health, Therefore, a review about health benefits of ALA from PS oil as compared to fish oil will be presented and discussed.

## 4. Selected Presentations

### 4.1. Proximate Analysis, Phytochemical Screening, Total Phenolic and Flavonoid Content, and Free Radical Scavenging Activity of the Philippine Bamboo “Buho” Schizostachyum lumampao Jovale Vincent Tongco, Ramon Razal, Remil Aguda

This research is a pioneering attempt to undertake proximate analysis, phytochemical screening, total phenolic and flavonoid content determination, and quantification of the free radical scavenging activity of a native bamboo species *Schizostachyum lumampao*, locally known as buho in the Philippines. Preliminary phytochemical screening of endemic plants is very important in drug discovery. It will lead to potential sources of bioactive compounds for health and beauty, specifically phenolics and flavonoids which are natural antioxidants. Proximate analysis showed that the air-dried buho leaves contain 10% moisture, 30.5% ash, 22.1% crude protein, 1.6% crude fat, 28.7% crude fiber, and 7.2% total sugar (by difference). Qualitative phytochemical screening detected saponins, diterpenes, triterpenes, phenols, tannins, and flavonoids in both the ethanolic and aqueous leaf extracts, while phytosterols were only detected in the ethanolic extract. Using the Folin-Ciocalteu method, the total phenolic content in gallic acid equivalent (GAE) per 100 g dried sample, was 76.72 ± 9.06 for the ethanolic extract and 13.48 ± 4.12 for the aqueous extract. The total flavonoid content in quercetin equivalent (QE), were 70.24 ± 7.52 and 17.86 ± 3.41 mg QE/100 g dried sample for the ethanolic and aqueous extracts, respectively. The antioxidant activity of different concentrations of buho ethanolic extract was determined using the DOPH (2,2-Di(4-*tert*-octylphenyl)-1-picrylhydrazyl) free radical scavenging activity (FRSA) assay. The % FRSA values for the ethanolic extracts were 51.27 ± 1.50, 52.33 ± 3.00, 54.98 ± 0.75, 56.57 ± 1.50 and 50.21 ± 0.00 for 10, 50, 100, 200 and 400 ppm, respectively. These initial results suggest the potential of buho as a natural health and nutrition supplement.

### 4.2. The Compositions of Omega-3 and Omega-6 Polyunsaturated Fatty Acid in Seven Sea Cucumber Species Sabreena Safuan

Sea cucumbers have been used in many communities worldwide because of its various medicinal potential. Despite the widespread used, research of this holothuroid is scarce and lots of bioactive *substances of sea cucumber believed to facilitate health and prevent diseases are yet to be identified*. Polyunsaturated fatty acids of the class omega-3 and omega-6 play an active role in inflammation and wound healing in which these sea cucumbers extract is widely used for. Therefore, the objective of this study is to determine the fatty acids composition of seven species of sea cucumbers namely *Sticopus hermanii*, *Sticopus chloronotus*, *Sticopus badionotus*, *Holothuria atra*, *Holothuria tubulosa* and two *Molpadiia* sp. using gas chromatography. Recorded values were expressed as mean ± standard deviation. Analysis of variances (ANOVA) was used to determine statistical significant. All species shows major differences in their polyunsaturated fatty acid compositions. *Holothuria atra* showed the highest percentage area of linoleic and linolenic acids which was 49.25% ± 2.73% and 23.16% ± 2.83% respectively. *Stichopus hermanii* showed the highest percentage area of arachidonic acid which was 26.73% ± 1.84%. Interestingly, the fatty acids composition of the two *Molpadiia* sp. taken from two different locations showed a significant different in term of their fatty acid profile. On the basis of this study, sea cucumbers could be used as a source of omega-3 and omega-6 fatty acids. The fatty acid compositions of *Molpadiia* sp. could be used as an indicator of scientific differentiation and classification of species.

### 4.3. Discovery and Development of a Potent Histone Deacetylase Inhibitor Derived from Marine Cyanobacteria Hendrik Luesch

Histone deacetylase (HDAC) inhibitors represent a relatively new class of anticancer agents that target dysregulated acetylation of histone lysines. Since HDACs also have non-histone targets, inhibitors of this enzyme class may additionally modulate protein-protein interactions affected by the acetylation status, which increases the potential biomedical utility of these epigenetic regulators. To date, two HDAC inhibitors—vorinostat and romidepsin—have reached the market, with romidepsin being a natural product and vorinostat closely related to the natural product trichostatin A. We discovered a potent class I HDAC inhibitor termed largazole, a cyclic depsipeptide from a marine cyanobacterium. Largazole possesses highly differential growth-inhibitory activity, preferentially targeting transformed over non-transformed cells. We solved the supply problem by total synthesis, defined structure-activity relationships, and explored opportunities to develop isoform specific inhibitors based on the largazole scaffold. Stability and pharmacokinetic studies suggested promising bioavailability of the active species, largazole thiol, which is released from the pro-drug largazole upon protein-assisted thioester hydrolysis. Our exploration of alternative pro-drug approaches led to altered activity profiles. Largazole strongly stimulated histone hyperacetylation in solid tumors and inhibited tumor growth. We also found that largazole cooperated with dexamethasone to induce E-cadherin localization to the plasma membrane in triple-negative breast cancers *in vitro* and *in vivo*, and to suppress cellular invasion. Largazole also showed *in vivo* bone-forming efficacy, extending its utility to bone-related diseases. Investigations into other applications are ongoing. The presentation will provide an overview from the initial discovery of largazole to latest mechanistic and *in vitro* and *in vivo* biological data.

### 4.4. Isolation and Identification of Potential Antineoplastic Bioactive Phenolic Compounds in Malaysian Honeys Norjihada Izzah Ismail*, Mohammed Rafiq Abdul Kadir, Razauden Mohamed Zulkifli

Role of honey as a cancer chemopreventive agent was strongly attributed from their phenolic composition. The present study aimed to isolate and identify phenolic compounds with anticarcinogenic potential from the sugar matrix of Malaysian honeys. Phenolic compounds in Malaysian Acacia, Gelam and Tualang honey samples were isolated using octadecyl (C18) silica solid phase extraction (SPE) technique and identification was performed using high-performance liquid chromatography (HPLC) with diode array detector (DAD). Identification of phenolic compounds was achieved by comparing chromatographic retention times of honey samples with those of authentic standard compounds. The HPLC analysis confirmed the presence of anticancer phenolic compounds in all honey samples with considerable variations observed for different types of honey and among same type of honey. Six flavonoids (quercetin, naringenin, kaempferol, rutin, hesperetin, and apigenin), two phenolic acids (*p*-coumaric acid, and ferulic acid) and two tannins (ellagic acid, and penta-*O*-galloyl-β-d-glucose [PGG]) were the bioactive anticancer compounds identified. The presence of PGG in Malaysian honey was described for the first time. This study concluded that these three types of Malaysian honey possessed anticancer properties at varying degree. Their potential usage as natural anticancer therapeutic agents with numerous health benefits could be further explored and considered as an alternative for current anticancer drugs.

### 4.5. The Cytotoxic Activity of Oligostilbenoidsandits Derivatives from the Stem Bark of Dryobalanops lanceolata against A549 and MCF-7 Cancer Cell Lines Agustono Wibowo*, Norizan Ahmat, Ahmad Sazali Hamzah

Isolation and purification of methanol extract from the stem bark of *D. rappa* yielded 14 oligostilbenoids. The structures of ɛ-viniferin (1), laevifonol (2), ampelopsin F (3), isoampelopsin F (4), ampelopsin E (5), malaysianols B (6) and C (7), nepalensinol B (8), hopeaphenol (9), flexuosol A (10), nepalensinol E (11), upunaphenol D (12), vaticanols B (13) and C (14)were established on the basis of spectroscopic evidences and comparison with published data. Selected compounds were further subjected to methylation and acetylation. The cytotoxic activity of all isolates, and its methyl/acetate derivatives (selected compounds) has been tested against A549 lung and MCF-7 breast cancer cell lines. Ampelopsin E (5) and vaticanol C (14) displayedmoderate activity against MCF-7 (IC_50_ 14.28 μg/mL) and A549 (IC_50_ 10.73 μg/mL) cell lines, respectively.

### 4.6. Dietary Fiber from Cassava Pulp Pornariya Chirinang*, Ratchadaporn Oonsivilai, Natta Kachenpukdee

Cassava pulp is a high value by-product from cassava starch industry that contain high amount of neutral detergent fiber (NDF) around 31.40% (w/w). Response surface methodology was applied for optimization of extraction parameters. Three parameters such as α-amylase concentration, protease concentration, and amyloglucosidase concentration including three levels for each parameter were studied. The optimum condition for the highest NDF preparation by enzymatic digestion was at 0.1% of α-amylase (w/v), 1% of protease (v/v) and 0.1% of amyloglucosidase (v/v). Dietary fiber from cassava pulp contained 40.24% (w/w) crude fiber, 79.03% (w/w) neutral detergent fiber (NDF), 70.14% (w/w) acid detergent fiber (ADF) and high content of cellulose at 58.55% (w/w). In addition, the hydration properties of dietary fiber prepared were investigated. The results showed that the dietary fiber prepared exhibited 4.82 mL/g swelling capacity, 8.36 g/g water retention capacity and 8.17 g/g water holding capacity. The major monosaccharide constituent of dietary fiber prepared was glucose, together with other neutral sugars. The FTIR spectrum of dietary fiber prepared was similar to cassava pulp spectrum with showing the sharp peak at 1005–1031 cm^−1^ that is usually the fingerprint of polysaccharides. Finally, scanning electron microscopy (SEM) of cassava pulp revealed a lot of starch granule embedded within cell wall material of cassava pulp. Otherwise, there was no starch granule appeared in dietary fiber prepared after enzymatic digestion. In conclusion, as physicochemical properties of dietary fiber prepared from cassava pulp by enzymatic digestion described above, cassava pulp could be used as a rich source of useful dietary fiber and could be applied to many food products.

### 4.7. New Sesquiterpenes from the Rhizome of Curcuma xanthorrhiza Roxb. and Their Inhibitory Effects on UVB-Induced MMP-1 Expression in Human Keratinocytes Ji-Hae Park*, Ye-Jin Jung, Seo-Ji In, Jung-Hwa Kwon, Byeong-Ju Cha, Sabina Shrestha, Mohamed Antar Aziz Mohamed, Tae Hoon Lee, Chang-Ho Lee, Daeseok Han, Jiyoung Kim, Nam-In Baek

The rhizome of *Curcuma xanthorrhiza* (turmeric spices, Zingeberaceae) has been used in Indonesia to exert diverse physiological effects for a treatment of liver problems and indigestion. Also, many clinical trials for human are underway, including multiple myeloma, pancreatic cancer, colon cancer, psoriasis, and Alzheimer’s disease. The active components in tuber of *C. xanthorrhiza* have been reported to be curcumins and several types of sesquiterpenes. Our previous experiment revealed the rhizome extracts to decrease MMP-1 expression in UVB-treated human keratinocytes. So, this study was initiated to isolate the anti-photoaging metabolites from the rhizome of *C. xanthorrhiza*. The dried and powdered rhizomes of *C. xanthorrhiza* were extracted with 80% aqueous MeOH, and the concentrated extract was partitioned with EtOAc, *n*-BuOH, and H_2_O, successively. The repeated silica gel, ODS, and Sephadex LH-20 column chromatographies for the EtOAc and *n*-BuOH fractions led to isolation of five curcuminoids and 19 sesquiterpenoids including three new compounds. From the results of spectroscopy data including NMR, IR, FAB/MS and EI/MS, they were identified to be five curcuminoids (1–5), nine bisabolane sesquiterpenes (6–14), six guaiane sesquiterpenes (15–20), and germacrane sesquiterpenes (21–24). Three new sesquiterpenes along with 12 known compounds were isolated for the first time from the rhizome of *C. xanthorrhiza* in this study. Compounds 10 and 14 decreased MMP-1 expression in UVB-treated human keratinocytes by about 8.9-fold and 7.6-fold at the mRNA level, and by about 9.2-fold and 6.6-fold at the protein level, respectively. The results indicate that the isolated compounds may have anti-aging effects through inhibition of MMP-1 expression in skin cells.

### 4.8. Isolation and Identification of Fourteen Ginsenosides from the Aerial Parts of Hydroponically Cultivated Panax ginseng C.A. Meyer, Byeong-Ju Cha*, Ji-Hae Park, Sabina Shrestha, Rak-Hun Jeong, Ye-Jin Jung, Nam-In Baek, Yong-Bum Kim, Myeong-Hun Yeom, Seung-Yu Kim, Dae-Young Lee

*Panax ginseng* C.A. Meyer is famous traditional medical plant. To date, many studies have been reported for the chemical constituents of ginseng cultivated in soil, more than 70 kinds of ginsenosides have been isolated. Currently, the interest in safe agricultural products of high quality is gradually increasing, leading to an increase in the cultivation acreage by hydroponics for the production of ginseng in high-tech cultural facilities. The hydroponically cultivation is very shorter than that of soil cultivation. The ginseng’s growth characteristics were examined in a harvesting time of maximum 120 days after transplanting the ginseng seedling roots into the aeroponic system. Several natural bioactive components of hydroponic *P. ginseng* were reported. In this study is conducted for isolation and identification of active metabolites from the aerial parts of hydroponic *P. ginseng*. The aerial parts of hydroponic *P. ginseng* were extracted with aqueous MeOH, and the concentrated extract was partitioned with EtOAc, *n*-BuOH, and H_2_O, successively. The repeated silica gel and ODS column chromatographies for EtOAc and *n*-BuOH fraction led to isolation of fourteen ginsenosides, ginsenoside F1 (1), ginsenoside F2 (2), ginsenoside F3 (3) ginsenoside F5 (4), ginsenoside Rh6 (5), floralginsenoside Ka (6), floralginsenoside A (7), floralginsenoside B (8), ginsenoside Rg1 (9), ginsenoside Rg2 (10), ginsenoside Rc (11), ginsenoside Rd (12), ginsenoside Re (13), and ginsenoside Rb2 (14). The chemical structures of 1-14 were determined by spectroscopic data interpretation. Supported by Next Generation Bio-Green 21 (PJ009544) Project from Rural Development Administration, Republic of Korea.

### 4.9. Flavonoids and Sterols from the Flowers of Begonia semperflorens Link et Otto. and Quantitative Analysis of Flavonoids and Anthocyanins Jung-Hwa Kwon*, Seo-Ji In, Kyeong-Hwa Seo, Ji-Hae Park, Jae-Woo Jung, Rak-Hun Jeong, Jin-Gyeong Cho, Nam-In Baek

“Flower” is the reproductive organ of the plant and has been used as an ornamental plant. In addition, flower’s constituents were reported as many secondary metabolites such as flavonoids, sterols, terpenes as well as pigment such as anthocyanins. And then, antioxidant, anti-inflammatory, cytotoxic and antibacterial activity have been reported. Rural Development Administration reported that edible flowers contain antioxidants ten times more than fruits and vegetables. *Begonia semperflorens* Link et Otto. (Begoniaceae), native to Brazil, is wide spread plants in tropical, subtropical region. This plant grows up into 15–45 cm and has broad oval leaves and white, pink, red flowers that bloom perennially. Among many edible flowers, begonia have been reported only about analysis of anthocyanins. In this study, authors report isolation and identification of six compounds from the flowers of *Begonia semperflorens* Link et Otto. The flowers of *Begonia Semperflorens* Link et Otto. (3 kg) were extracted with 80% aqueos MeOH, and the concentrated extract was partitioned with EtOAc, *n*-BuOH, and H_2_O, successively. From the EtOAc fraction, four flavonoids and two sterols were isolated through the repeated SiO_2_, ODS column chromatographies. According to the results of spectroscopic data including NMR, MS and IR, these compounds were identified to be four flavonoids, astragalin (1), isoquercetin (2), kaempferol (3), quercetin (4) and two sterols, β-sitosterol (5) and daucosterol (6). Compounds **1**–**6** were isolated for the first time from the flowers of *Begonia semperflorens* Link et Otto. Furthermore, flavonoids and anthocyanis were analyzed using HPLC and LC-MS experiments, respectively.

### 4.10. Secondary Metabolites from the Root Bark of Morus alba L. Jae-Woo Jung*, Ji-Hae Park, Ye-Jin Jung, Jung-Hwa Kwon, Kyeong-Hwa Seo, Jin-Gyeong Cho, Chang-Ho Lee, Daeseok Han and Nam-In Baek

The *Morus alba* L. (Moraceae) is widely distributed in Korea, Thailand, China and Japan. Its root bark (Sang-Bai Pi) is Chinese traditional medicine widely used for treating diabetics, relieving asthma and protecting liver. Previously, some researchers reported the chemical constituents of the mulberry root bark as isoprenyl flavonoids, stilbenes, coumarins, and benzofurans. These compounds are reported to show anti-oxidant, anti-inflammatory, anti-cancer and anti-microbial activities. Therefore, our studies focused on isolation and identification of pharmacological compounds from the mulberry root bark. The dried and powdered mulberry root bark was extracted with 80% aqueous MeOH, and the concentrated extract was partitioned with EtOAc, *n*-BuOH, and H_2_O, successively. The repeated silica gel and ODS column chromatographies for EtOAc and *n*-BuOH fractions led to isolation of sixteen compounds. From the result of spectroscopic data including NMR, HPLC, Prep-LC, FAB/MS and EI/MS, the structure of these compounds were identified as two new isoprenyl flavonoids (1,2), sanggenol A (3), sanggenol O (4), sanggenol P (5), sanggenol L (6), sanggenon F (7), sanggenon G (8), sanggenon N (9), sanggenon I (10), moracin O (11) and P (12), two new moracin diglucoside (13,14). mulberrofuran G (15) and mulberrofuran C (16). Compounds **1**–**2**, **4**, **11**–**14** were isolated for the first time from the mulberry root bark.

### 4.11. Chemical Constituents and Antimicrobial Properties of the Essential Oil and Ethanol Extract from the Stem of Aglaia odorata Nantiya Joycharat*, Supayang Piyawan Voravuthikunchai, Patimaporn Plodpai, Sonesay Thammavong, Watcharapong Mitsuwan, Sanan Subhadhirasakul

The essential oil and ethanol extract from the stem of *Aglaia odorata*, collected from Southern Thailand, were investigated for their chemical components and antimicrobial activity. The stem-derived oil of *A. odorata* was obtained by hydrodistillation using a Clevenger type system. Gas chromatography–mass spectrometry analysis of the oil revealed the identification of 43 compounds, representing 82.53% of the oil; aromadendrene (21.73%), α-himachalene (18.27%), valencene (13.64%), and β-caryophyllene (10.96%) were the major components. Antimicrobial activities of the oil and ethanol extract were tested against two Gram-positive and three Gram-negative bacteria including *Bacillus cereus* ATCC 11778, *Staphylococcus aureus* ATCC 25923, *Acenetobacter baumannii* ATCC 19606, *Escherichia coli* ATCC 25922, and *Pseudomonas aeruginosa* ATCC 10145, as well as three rice fungal pathogens *Bipolaris oryzae*, *Pyricularia oryzae*, and *Rhizoctonia solani* using broth microdilution method. The oil exhibited significant antimicrobial activity against five of eight pathogens tested, particularly the three rice pathogens (MIC 0.0625–0.5 mg/mL). The ethanol extract and its one main component isolated, eichlerialactone, showed activity against all the fungal pathogens tested (MIC 0.25–2 mg/mL). Besides the antifungal activity, eichlerialactone exhibited good antibacterial activity against both of Gram-positive pathogens tested (MIC 0.25–0.5 mg/mL) as well.

### 4.12. Inhibition of Tyrosinase Activity by Polyphenol Compounds from Flemingia philippinensis Roots Yeong Hun Song*, Wang Yan, Won Min Jeong, Ki Hun Park

*Flemingia philippinensis* is used as a foodstuff or medicinal plant in the tropical regions of China. The methanol (95%) extract of the roots of this plant showed potent tyrosinase inhibition (80% inhibition at 30 µg/mL). The production of melanin by tyrosinase is essential for the protection of skin from solar radiation. Tyrosinase also serves a protective function in plants and is responsible for browning of damaged fruits during post-harvest handling and processing. Acivity-gudied isolation yield six polyphenols that inhibited both the monophenolase (IC_50_ = 1.01–18.4 µM) and diphenolase (IC_50_ = 5.22–84.1 µM) actions of tyrosinase. Compounds **1**–**6** emerged to be three new polyphenols and three known flavanons, flemichin D, lupinifolin and khonklonginol H. The new compounds (**1**–**3**) were identified as dihydrochalcone which we named fleminchalcones (**A**–**C**), respectively, The most potent inhibitor, dihydrochalcone (3) showed significant inhibitions against both the monophenolase (IC_50_ = 1.28 µM) and diphenolase (IC_50_ = 5.22 µM) activities of tyrosinase. Flavanone (4) possessing a resorcinol group also inhibited monophenolase (IC_50_ = 1.79 µM) and diphenolase (IC_50_ = 7.48 µM) significantly. In the kinetic studies, all isolated compounds behaved as competitive inhibitors. Fleminchalcone A was found to have simple reversible slow-binding inhibition against monophenolase.

### 4.13. Flavonoids from Campylotropis hirtella Displaying α-Glucosidase Inhibition Xuefei Tan*, Jeong Yun Kim, Ki Hun Park

The α-glucosidases (EC 3.2.1.20) are a group of exacting enzymes that play essential roles in carbohydrate metabolism and in glycoprotein processing. Especially, these enzymes play a critical role on modification of glycoprotein structure, which consequently affects the maturation, transport, secretion and function of glycoprotein to alter cell-cell or cell-virus recognition process. Thus, α-glucosidases inhibition is applicable to treatment of numerous disease including diabetes mellitus type II, cancer, and HIV. *Campylotropis hirtella* belongs to the plant family Leguminosaea. Its roots are traditionally used in Chinese folklore to treat disease such as irregular menstruation, dysmenorrheal, metrorrhagia and gastric ulcers. In continuous search of glycosidase inhibitors, the methanol extract of the roots of *Campylotropis hirtella* showed high α-glucosidases inhibitory activity with an IC_50_ of around 100 μg/mL. Due to its potency, subsequent bioactivity-guided fractionation of methanol extract led to 2ʹ-methoxy-6,3ʹ-diprenyl-6,8,4ʹ-trihydroxyisoflanone, 3ʹ-(3,7-dimethylocta-2*E*,6-dien-1-yl)-5,7,4ʹ-trihydroxyisoflavone, 5,7-dihydroxy-3-[2,4-dihydroxy-3-(3-methyl-2-butenyl)phenyl]-6-(3-methyl-2-butenyl)-2,3-dihydro-4*H*-1-benzopyran-4-one-trihydroxyisoflanone, 3,5,7,4ʹ-tetrahydroxy-6,3ʹ-diisopropyl-2ʹ-methoxy-isoflavanone, 2*S*-3ʹ-(3,7-dimethylocta-2*E*,6-dien-1-yl)-6-methyl-5,7,4ʹ-trihydroxyflavanone, and (2*R*,3*R*)-6-methyl-3ʹ-geranyl-2,3-trans-5,7,4ʹ-trihydroxyflavonol. These compounds (**1**–**6**) were evaluated for α-glucosidase inhibitory activity to identify their inhibitory potencies and kinetic behavior. The active components were identified as flavonoid derivatives consisting of flavones dihydroflavanols and isoflavones that inhibited α-glucosidase a dose-dependent mauner, with IC_50_’s ranging between 18 and 130 µM. The most potent inhibitor was found to be (2*R*,3*R*)-6-methyl-3ʹ-geranyl-2,3-*trans*-5,7,4ʹ-trihydroxyflavonol with IC_50_ of 18.3 μM. The all isolated inhibitors (**1**–**6**) are similar in activity to sugar derived α-glucosidase inhibitors as voglibose (IC_50_ = 23.4 μM). In kinetic studies, all compounds (**1**–**6**) exhibited mixed typed inhibition characteristics.

### 4.14. The Plausible Biogenetic Pathway of Oligostilbenoids and Its Chemotaxonomic Significance on the Placement of Dryobalanops in the Dipterocarpaceae Family Norizan Ahmat*, Agustono Wibowo

*Dryobalanops* (Dipterocarpaceae) is a major species in emergent canopy in Lambir Forest and generally in Sarawak lowland Dipterocarp forest. As other genus in Dipterocarpaceae family, *Dryobalanops* have been shown to be a rich source of oligostilbenoids. However, the different skeleton-types of oligostilbenoids in each family or genera have attracted interests from scientists of various disciplines to investigate their properties such as phytochemical constituents, biogenetic pathway and chemotaxonomy. The skeleton of oligostilbenoids from *Dryobalanops* is unique due to the unusual condensation types compared to other Dipterocarpaceae species. In this study, seventeen oligostilbenoids have been isolated from the stem bark of four *Dryobalanops* species, that are five dimmers; ε-viniferin (1), diptoindonesin A (2), laevifonol (3), ampelopsin F (4) and isoampelopsin F (5), three trimers; malaysianol A (6), ampelopsin E (7) and α-viniferin (8), and nine tetramers; malaysianol B (9), nepalensinol B (10), hopeaphenol (11), stenophyllol A (12), flexuosol A (13), nepalensinol E (14), upunaphenol D (15), vaticanols B (16) and C (17). Their structures were established on the basis of spectroscopic evidences and comparison with published data. Biogenetically, the condensation types of all isolated oligostilbenoids are typical for dipterocarpaceous plant, with the exception of 6 and 15 that are formed from C7-C14 and C8-C3 type of condensation, respectively. Based on the recent molecular phylogeny studies, *Dryobalanops* was placed as intermediate between the tribes Dipterocarpeae and Shoreae. However, our phytochemical findings, showed close relation of *Dryobalanops* to the tribe Dipterocarpeae rather than Shoreae.

### 4.15. Identification of New Lipo-Alkaloids from Aconiti Radix by UHPLC-Q-TOF-MS/MS Approach Na Li*, Ying Liang, Jian-Lin Wu

Aconiti Radix is widely used as anti-inflammatory and analgesic agent in Eastern Asia. Lipo-alkaloid is a type of alkaloids in *Aconitum* species, which usually contains an aconitane skeleton and one or two long-chain fatty acid residues. Due to the high similarity of the structures, it’s difficult to obtain the pure lipo-alkaloids. Ultra-High Performance Liquid Chromatography-Quadrupole-Time of Flight-mass spectrometry (UHPLC-Q-TOF-MS) approach can provide excellent separation and structural elucidation, and thus was used to identify the lipo-alkaloids in Aconiti Radix in our research. The high resolution MS and characterizations of fragmentation ions in MS/MS were firstly investigated. Then, 46 lipo-alkaloids were identified based on their MS and MS/MS spectra, and 6 new compounds were identified as 14-benzoylhypaconine-8-*O*-docosatetrenoate, 14-benzoylhypaconine-8-*O*-heneicosahexenoate, 14-benzoylmesaconine-8-*O*-heneicosahexenoate, 14-benzoylmesaconine-8-*O*-docosapentenoate, 14-benzoylmesaconine-8-*O*-heneicosapentenoate, and 14-benzoylmesaconine-8-*O*-octadecatetrenoate, respectively, using this approach.

### 4.16. Effects of Polar Fraction of Punica granatum L. on Body Weight Gain and Total Cholesterol of Osteoporotic Model Rat Ade Arsianti*, Nurul Qomariyah, Anton Bahtiar

The effects of polar fraction of *Punica granatum* (L.) on body weight gain and total cholesterol of ovariectomized (ovx) rat model of osteoporosis were investigated. Forty-two 6-weeks-old female Sprague–Dawley rats were randomly assigned to seven groups as followed, sham-operated, OVX, OVX-Estradiol (0.5 mg/kgBW), OVX-Tamoxifen (50 mg/kgBW), OVX-Punica fraction (PF) 50 mg/kgBW, OVX-PF 100 mg/kgBW and OVX-PF 200 mg/kgBW for 4 weeks. The administration of Punica fraction was given orally using a stomach tube. The results demonstrated that the administration *Punica* fraction 50, 100, and 200 mg/kg BW significantly prevented body weight gain compared with ovariectomy rat. The decreasing of body weight gain was dose dependent and at 200 mg/kg BW showed better in decreased of body weight. And the effect same as tamoxifen control rat. Total cholesterol serum also showed decreasing amount of total cholesterol compared with the ovariectomy rat. This study suggest that polar fraction of *Punica granatum* L. extract may offer a potential alternative therapy for the treatment of health problems in obesity.

### 4.17. Effects of Ellagic Acid on Enhancing Mucosal Immune Mediators and HIV-1 Infection Inhibition Aornrutai Promsong*, Florian Hladik, Thippawan Chuenchitra, Wipawee Nittayananta

Mucosal epithelial cells are major portal for human immunodeficiency virus type 1 (HIV-1) transmission. Some antimicrobial peptides such as human β-defensin 2 (hBD2) and secretory leukocyte protease inhibitor (SLPI) were expressed by the epithelial cells and have been shown their anti-HIV-1 activities. Enhancement of these protein expressions could promote the innate immunity. In this study, a plant-based anti-integrase ellagic acid (EA) was discovered as a new agent to induce the expression. This present work aimed to determine the enhancement of immune mediators expression in human vaginal epithelial cells (HVEs) by EA and its inhibitory effect against HIV-1 infection in C8166 and TZM-b1 cells. EA at a concentration ranging from 1.56 to 100 µM was used to assess the activities. After the primary HVEs had been treated with EA for 18 h, the immune mediators were measured using ELISA and Luminex assay. To verify the anti-HIV-1 activity, the C8166 and TZM-bl cells were treated with EA before and after they were infected. The HIV-1 replications were then assessed using p24 ELISA and Nano-Glo-Luciferase assay. For immune mediators study, a dose-dependent increase in hBD2, SLPI, and IL-2 expressions were significantly found in EA group. Subject to the inhibitory effect, EA suppressed HIV-1 replication for up to 67% before and after the cells had been infected in both cell lines. In conclusion, this study showed a potency of EA for inhibiting HIV-1 activities and inducing the immune mediators. The results could be applied for topical drug development in the future.

### 4.18. Hepato-Protective Effect of Azadirachta indica Leaf Aqueous Extract against Plasmodium berghei Infected Mice Voravuth Somsak*, Ubonwan Jaihan, Somdet Srichairatanakool, Chairat Uthaipibull

Malaria is caused by protozoa parasite in genus *Plasmodium* and transmitted by *Anopheles* mosquito. It is estimated 1 million deaths annually, most of them are children less than 5 years of age in sub-Saharan Africa. Causes of death in malaria are variable including severe anemia, cerebral malaria, and liver damage and failure. Especially in liver damage during malaria infection is a major focus of this study. The objective of this study was to determine protective effect of *Azadirachta indica* leaf extract against *Plasmodium berghei*-induced liver damage by using aspartate and alanine aminotransferase (AST and ALT, respectively) as biological markers. Aqueous leaf extract of *A. indica* was prepared. For *in vivo* test, ICR mice were inoculated with 1 × 10^7^ infected red blood cells of *P. berghei* ANKA. The extract (1000 mg/kg) was orally given twice a day for 6 consecutive days, and AST and ALT were then measured using commercial kits. It was found that AST and ALT levels in plasma were significantly (*p* < 0.05) increased on day 6 post infection resulting to parasite development *in vivo*. Interestingly, infected mice treated with this extract, AST and ALT levels were normalized significantly and no difference to normal mice. It can be concluded that *A. indica* leaf extract exerted protective effect on liver damage during malaria infection. However, active components and mechanism of action should be studied in more detail for validating this extract as alternative malaria treatment.

### 4.19. Screening and Characterization of Indigenous Lactic Acid Bacteria against Malathion Induced Toxicity Using Model Organism Caenorhabditis elegans Arumugam Kamaladevi, Krishnaswamy Balamurugan*

Malathion an organophosphorous insecticide is renowned to cause neurotoxicity primarily by inhibiting acetylcholinesterase (AChE), an excellent biomarker of OP exposure. Inhibition of AChE is widely lead to widespread disturbance in the normal physiological and behavioral activity of an organism. Lactic acid bacteria (LAB) are still an underexploited and inexhaustible source of significant pharmaceutical thrust. In the present study *Caenorhabditis elegans* has been employed to identify and characterize the novel LAB isolated from different traditional food against malathion induced toxicity. Different physiological and behavioral assays were performed for the identification of LAB that promotes the survival of *C. elegans* against malathion. Among 4 selectively screened strains, *L. casei* and *L. bulgaricus* displayed excellent rescuing ability in malathion exposed *C. elegans*. Short term exposure assay concluded that *L. casei* isolated from mango pickle acts as a better food for survival during toxication than *L. bulgaricus*. Further, the protective potential of the *L. casei* has been investigated against malathion induced AChE inhibition. Interestingly, pretreated nematodes exposed to malathion significantly (*p* < 0.05) reduced the inhibition of AChE to 1.25%, whereas malathion showed 37% of inhibition. The reduced inhibition of AChE in pretreated nematode was accompanied with rescued life span, reproduction, feeding and locomotion. Malathion required 36 h for complete killing of *C. elegans* at LD_50_, while pretreatment with *L. casei* significantly (*p* < 0.05) enhanced their survival to 14 days. Additionally, pretreatment effectively rescued egg laying, brood size, pharyngeal pumping, feeding and locomotion in malathion exposed animals. Thus the overall results suggested the protective effect of the *L. casei* against malathion is mediated through rescuing the AChE inhibition and hence the present study reports for the first time that an indigenous LAB-*L. casei* could act as a protective agent against the harmful effect of pesticide.

### 4.20. Free Radicals Scavenging and Anti-Oxidative DNA Damage Activities of Active Fractions from Lansium domesticum Corr. Fruit Prapaipat Klungsupya*, Nava Suthepakul, Thanchanok Muangman, Sarunya Laovitthayanggoon, Jeerayu Thongdon-A, Srisak Trangwacharakul

“Long-kong” *Lansium domesticum* Corr. belongs to the family Meliaceae. In this study, skin (SK) and seeds (SD) of mature long-kong fruits were extracted using 50% and 95% ethanol. The four ethanolic extracts were then partitioned between dichloromethane (DCM) and 50% aqueous ethanol. The obtained aqueous phase (H_2_O) was further extracted with ethyl acetate (EA). The partition procedure yielded 12 fractions namely; LDSK50-DCM, LDSK50-EA, LDSK50-H_2_O, LDSK95-DCM, LDSK95-EA, LDSK95-H_2_O, LDSD50-DCM, LDSD50-EA, LDSD50-H_2_O, LDSD95-DCM, LDSD95-EA and LDSD95-H_2_O. The 12 long-kong fractions were subjected to photochemiluminescence (PCL) and 2-deoxyribose (2-DR) assays to determine their anti-oxidant capacity on superoxide anion radical (O_2_^−^^•^) and hydroxyl radical (OH^•^) scavenging activities, respectively. Subsequently, the active fractions were assayed for their anti-oxidative activity against DNA damage by hydrogen peroxide (H_2_O_2_) in human TK6 cells (ATCC CRL-8015) using the comet assay. Results of the PCL and DR assays demonstrated that fractions LDSK50-EA and LDSK50-H_2_O were most potent against O_2_^−^^•^ and OH^•^ radicals. The comet results revealed DNA-protective activity of both LDSK50-EA and LDSK50-H_2_O fractions when TK6 cells were pre-treated at 25, 50, 100 and 200 μg/mL for 24 h prior to exposure to H_2_O_2_. LDSK50-EA could prevent DNA damage in a dose-dependent manner and the highest effect found at 200 µg/mL concentration. This study generates new information on biological activity of *L. domesticum* Corr. fruits that has not yet been reported before. The anti-oxidant capacity against O_2_^−^^•^ and OH^−^^•^ radicals and cellular DNA protective activity against H_2_O_2_ will promote and strengthen utilisation of *L. domesticum*.

### 4.21. Artesunate Attenuates Tumorigenesisin Transgenic Adenocarcinoma of Prostate (TRAMP) Mice by Modulating Pro-Inflammatory Pathways Muthu K. Shanmugam*, Tina H. Ong, Amudha deivasigamani, Alamelu Nachiyappan, Lou Wenxia, Kong Sing Tiang, Paul C. Ho, Kam Man Hui, Gautam Sethi

Prostate cancer incident rates have risen rapidly worldwide and the increased incidence can be associated with both genetic susceptibility and exposure to distinct environmental factors. Hence there is an urgent need to discover novel compounds for the treatment of prostate cancer. In this study, we evaluated the potential effect of a semi-synthetic derivative of artemisinin, artesunate (50 mg/kg b.w., oral gavage) on the initiation and progression of prostate cancer in a transgenic adenocarcinoma of prostate (TRAMP) mouse model. TRAMP mice were divided into three groups based on their age and duration of feeding to evaluate the chemopreventive activity against prostate cancer. We found that in group 1, four week old mice fed with artesunate for 8 weeks delayed the formation of prostate intraepithelial neoplasia (PIN). In group 2, eight week old mice were fed with artesunate for 6 weeks delayed the progression of PIN to adenocarcinoma as determined by hematoxylin and eosin staining. In group 3, twenty four week old mice were fed with artesunate for 12 weeks significantly inhibited tumor growth and tumor metastasis to lungs and liver without any drastic changes in body weight and prolonged the survival of mice. When investigated for the molecular mechanisms, we found that artesunate downregulated various proinflammatory mediators in the dorsolateral prostate (DLP) tissue and down regulated the serum levels of major inflammatory molecules such as TNF-α and IL-6. Immunohistochemical analysis of DLP tumor tissues revealed that artesunate significantly downregulated cyclin D1, COX2, and increased the levels of caspase-3. Finally, we also determined the systemic bioavailability of artesunate in serum samples obtained from various artesunate fed mice groups. Artesunate was detected in the serum samples and was in microgram quantities. In conclusion our findings provide strong evidence that artesunate is well absorbed in the gastrointestinal tract and can be used as a drug for both prevention and therapy of prostate cancer.

### 4.22. Mechanism of the Long Lasting Vasorelaxant Effects of Apocynum venetum: Interplay between Nitric Oxide Release and Free Radical Scavenging Mustafa MR*, Lau YS and Dharmani M, CYKwan

The present study seeks to investigate the interplay between nitric oxide (NO) and superoxide anions (SOA) in the mechanism of the long lasting vasorelaxant effects of *Apocynum venetum* (AVLE) or Luobuma, a traditional Chinese medicine used for hypertension. The vasorelxant effects of AVLE on phenylephrine (PE)-induced contraction was assessed in rat aortic rings. Total nitrite/nitrates level was accessed by Griess reagent. Superoxide anion (SOA) production was measured by using enhanced-chemiluminescence assay. Primary rat aorta endothelial cells (RAEC) were used to examine the protein expression of the phosphorylation level of eNOS (endothelial nitric oxide synthase) and total eNOS in the presence of AVLE. In the rat aorta, AVLE (0.3–10 µg/mL) dose-dependently inhibited the contraction to PE (0.1 μM) and significantly suppressed the β-NADPH-induced generation of SOA. Removal of endothelium and treatment with l-NAME, prevented the vasorelaxant effects of AVLE. In addition, AVLE increased the total nitrite and nitrate (NOx) from 7.0 ± 4.3 to 43.0 ± 17.5 µM/mg protein. Repeated washout of AVLE for up to 90 min failed to reduce the NO level nor restore the responses to PE. In the RAEC, AVLE caused a dose-dependent increased of p-eNOS activation. The results suggest that the long lasting vasorelaxant effect of AVLE is due to its ability to sustain NO effect following the release and it has a superoxide scavenging property all of which account for its unique effect.

### 4.23. 6-Shogaol, a Potential Neuritogenic Compound that Mimics the Neurite Outgrowth Activity of Nerve Growth Factor Hong Sok Lai*, Syntyche Seow Ling Sing, Vikineswary Sabaratnam, Lee Guan Serm, Sri Nurestri Abd Malek

Neurotrophin, a nerve growth factor (NGF), has potent biological activities, such as promoting neuronal survival, proliferation and differentiation. However, it may not be effective in therapy because it is a high molecular weight polypeptide (which is unable to cross the blood-brain barrier). The aim of this study was to investigate the potential of 6-shogaol to mimic NGF activity in promoting neurite outgrowth. 6-Shogaol was isolated from the ethyl acetate extract of the rhizomes of *Jahe gajah* (*Zingiber officinale* var *officinale*) obtained from Yogyakarta, Indonesia. The rat pheochromocytoma (PC-12) cell line was used as an *in vitro* model. Nerve growth factor (50.0 ng/mL) was used as positive control. All the concentrations of 6-shogaol tested significantly (*p* < 0.05) promoted neurite outgrowth in PC-12 cells compared to the negative control. Maximum stimulation of neurite outgrowth was achieved at 500.0 ng/mL and was significantly (*p* < 0.05) higher compared to NGF. There was no detectable cytotoxic effect in the cells. The present findings suggested that 6-shogaol mimics the neurite outgrowth activity of NGF and may promote and maintain neuronal health.

### 4.24. Effect of Acetone Extract of Culinary Mushroom Pleurotus ostreatus on Carrageenan Induced Phagocytic Cell Infiltration and Nitric Oxide Production by Rat Peritoneal Cells WJA Banukie N Jayasuriya*, Shiroma M Handunnetti, Chandanie A Wanigatunga, Gita H Fernando, Tusitha U Abeytunga, T Sugandhika Suresh

This study investigates the effects of acetone extract (AE) of *Pleurotus ostreatus* (*P. ostreatus*) on carrageenan induced infiltration of cells and nitric oxide (NO) production of rat peritoneal cells. Three groups of rats (*n* = 6/group) were orally treated with AE of *P. ostreatus* (500 mg/kg), prednisolone (10 mg/kg) and distilled water. After 1 h, carrageenan (5 mg/kg) was injected into the peritoneal cavity followed by sterile phosphate buffered saline (40 mL) 2 h later. Peritoneal fluid (35 mL) was drained five minutes later, centrifuged and phagocytic/macrophage cell counts were done. Peritoneal cells collected from each rat were cultured at 1 × 10^6^ cells/mL. After 24 h, culture supernatant was assessed for *in vivo* NO production, using Griess reagent. For *in vitro* assay, peritoneal cells were collected from rats not exposed to extract *in vivo* and then treated *in vitro* with 3.90, 7.81, 15.62, 31.25, 62.50 and 125.00 μg/mL of AE of *P. ostreatus* for 30 min at 37 °C. Cells were cultured for 24 h and NO production was assessed. Oral treatment with AE of *P. ostreatus* and prednisolone significantly inhibited the infiltration of phagocytic cells (45.4% ± 5.6% and 61.3% ± 2.2% reduction; *p* < 0.01 respectively) and NO production (91.2% ± 1.3% and 95.6% ± 0.2% reduction; *p* < 0.001 respectively). *In vitro* assay demonstrated a dose-dependent inhibition of NO production (*r* = 0.95; *p* < 0.05). The dose of 125.00 μg/mL showed maximum inhibition (76.9% ± 2.9%). Inhibition of cell migration to the site of inflammation and NO production may contribute as possible mechanisms of anti-inflammatory activity of *P. ostreatus*.

### 4.25. Effects of Tephrosia purpurea and Momordica dioica on Streptozotocin-Induced Diabetic Nephropathy in Rats Avijeet Jain*, Alok Nahata, Santram Lodhi, Abhay K Singhai

Present study was aimed to investigate the effects of *Tephrosia purpurea* (TP) and *Momordica dioica* (MD) in streptozotocin-induced diabetic nephropathy in wistar rats. Rats were divided into 12 groups (*n* = 6). Diabetes was induced with a single dose of streptozotocin (55 mg/kg i.p.). Day 4 of the study was considered first day of the study as administration of ethanolic extracts of TP leaves and MD fruits were started on 4th day. In protective regimen (initial 26 days of experimental period), ethanolic extract was administered orally at 200 and 400 mg/kg/day, whereas curative regimen began after 26 days and continued up to 40 days. Diabetic control group was considered as standard for comparison. Serum glucose, urea, creatinine and urine albumin levels were significantly lower in treated groups as compared to the diabetic control. Malondialdehyde was significantly (*p* < 0.001) lowered in treated groups as compared to diabetic control group. Treated groups have shown significant increase (*p* < 0.001) in reduced glutathione level which was reduced in diabetic control group. Change in body weight was found to be non-significant in treated groups. It is concluded that TP leaf and MD fruit extracts can provide a radical cure for drug-induced diabetic nephropathy by a reduction in renal damage.

### 4.26. Antihyperglycemic Effects of Methanol Extract of Syzygium polyanthum Leaves in Streptozotocin-Induced Diabetic Rats Tri Widyawati*, Nor Adlin Yusoff, Mohd. Zaini Asmawi, Mariam Ahmad

*Syzygium polyanthum* (*S. polyanthum*), a plant belonging to Myrtaceae is widely used in Indonesian and Malaysian cuisine. In addition, it is also commonly used traditionally by diabetic patients in Indonesia. Hence, this study was conducted to investigate the hypoglycemic and antihyperglycemic effects of methanol extract of *S. polyanthum* leaves and the possible mechanism of action. To test for hypoglycemic activity, the methanol extract of *S. polyanthum* was administered orally to normal male Sprague Dawley rats after a 12 h fasting. To further test for antihyperglycemic activity the same treatment was administered to glucose loaded (intraperitoneal glucose tolerance test, IPGTT) and streptozotocin (STZ)-induced diabetic rats, respectively. Hypoglycemic test in normal rats did not show significant reduction in blood glucose levels by the extract. Furthermore, IPGTT conducted on glucose loaded normal rats also did not cause significant lowering of blood glucose. However, antihyperglycemic test conducted in STZ-induced diabetic rats showed significant reduction at the seventh hour after treatment (*p* < 0.01). A short-term (6-days) treatment was performed, whereby the diabetic rats were treated with the extract (treated group), metformin (positive control group) and normal saline (negative control group). The results demonstrated that the extract and metformin significantly reduced blood glucose levels by 62.7% and 51.3%, respectively compared to control group. The possible antihyperglycemic mechanisms of *S. polyanthum* were assessed by measurement of glucose absorption from the intestine and glucose uptake by isolated rat abdominal muscle. It was found that the extract not only increased glucose absorption from the intestine but also significantly increased its uptake by muscle tissues. A preliminary phytochemical qualitative analysis indicate the presence of tannins, glycosides, flavonoid, alkaloid and saponin. GCMS analysis indicated that methanol extract of *S. polyanthum* contains squalene. In conclusion, *S. polyanthum* methanol leaves extract exerted its antihyperglycemic effects possibly by inhibiting glucose absorption from intestine and promoting glucose uptake by muscles.

### 4.27. Antihyperglycemic Effects of Umbelliferone in High-Fat Diet/Streptozotocin-Induced Type 2 Diabetic Rats Jarinyaporn Naowaboot*, Nuntiya Somparn, Supakate Saentaweesuk, Supaporn Vannasiri

Umbelliferone (UMB) is a natural product of coumarin family. In this study, the antihyperglycemic effect of UMB on high-fat diet (HFD) and streptozotocin (STZ)-induced type 2 diabetes in Wistar rats was determined. Diabetes was induced by feeding 45% HFD for four weeks followed by a single injection of STZ (35 mg/kg, intraperitoneally). Normal control rats were treated with 5% gum arabic and diabetic rats were treated with UMB (10, 30 and 60 mg/kg) or 5% gum arabic or pioglitazone (10 mg/kg) for eight weeks. Fasting blood glucose (FBG), oral glucose tolerance test (OGTT), insulin and adiponectin were measured. Results indicated that UMB (10 and 30 mg/kg) significantly (*p* < 0.05) reduced blood glucose, and improved the OGTT. Moreover, the levels of insulin and adiponectin were increased after 30 mg/kg UMB treatment. These results suggest that UMB is effective in reducing hyperglycemia and improving insulin sensitivity in chronic diabetes. Therefore, it may have a therapeutic value for the treatment of type 2 diabetes.

### 4.28. Ferulic Acid Alleviates Metabolic Syndrome in High-Carbohydrate, High-Fat Diet Fed Rats Ketmanee Senaphan*, Orachorn Boonla, Upa Kukongviriyapan, Poungrat Pakdeechote, Veerapol Kukongviriyapan, Patchareewan Pannangpetch

Metabolic syndrome (MS) is a cluster of metabolic abnormalities characterized by obesity, high fasting glucose, hypertension, dyslipidemia, chronic low-grade inflammation and oxidative stress. Naturally antioxidant compounds could be a cost-effective intervention to reverse these changes. Chronic feeding of a diet with high carbohydrate and high saturated fats (HCHF) induce MS in experimental animals. Ferulic acid (FA) is the major phenolic compound found in rice oil and various fruits and vegetables. In this study, we tested the hypothesis that administration of FA reduces MS induced by HCHF diet. Male Sprague Dawley rats were randomly divided into three groups (*n* = 8/group). Rats in group 1 were fed with standard chow. Rats in groups 2 and 3 were fed with a HCHF diet and 15% fructose in drinking water for 16 weeks. After receiving HCHF diet for 10 weeks, rats in group 3 were orally administered with FA (30 mg/kg) for the last 6 weeks. Rats fed with HCHF diet showed the symptoms of MS. FA at tested dose significantly reduced blood pressure, fasting blood glucose and plasma triglyceride levels, whereas plasma HDL-C level was increased in HCHF fed rats (*p* < 0.05). Additionally, plasma malondialdehyde and vascular superoxide production were decreased, while the concentration of plasma nitric oxide (NO) metabolites was increased in MS rats treated with FA. These findings demonstrate that FA can exert the hypotension, hypoglycemic and hypolipidemic effects through the mechanisms that may be associated with increasing NO bioavailability and reducing oxidative stress in HCHF diet fed rats.

### 4.29. Salacinol and Related Analogs, New Leads for Type 2 Diabetes Therapeutic Candidates from Thai Traditional Natural Medicine Salacia chinensis Toshio Morikawa*, Junji Akaki, Kiyofumi Ninomiya, Eri Kinouchi, Genzoh Tanabe, Masayuki Yoshikawa, Osamu Muraoka

Genus *Salacia* (Hippocrateaceae), climbing shrubs widely distributed in Thailand, Sri Lanka, India, and Southeast Asian countries, have traditionally been used for treatment of diabetes in traditional medicine. A hot water extract from the stems of *S. chinensis* was found to significantly suppress the increase of postprandial blood glucose levels in α-starch loading rats in a dose-dependent manner (30–300 mg/kg, p.o.). Administrations of 0.25% and/or 0.50% of this extract containing dietary feeds for 3 weeks in KK-A^y^ mice were found to significantly suppress both blood glucose and HbA1c levels without significant changes of the body weight and food intake. The mechanism of action was revealed to be α-glucosidase inhibition, and through the bioassay-guided separation by monitoring the inhibitory activity against rat small intestinal α-glucosidase, we isolated several novel thiosugar sulfonium inner salts and their desulfonated analogs, e.g., salacinol (1), kotalanol (2), neosalacinol (3), and neokotalanol (4). These potent enzymatic inhibitory activities were also observed in human α-glucosidase (IC_50_ = 3.9–9.0 μM for maltase). These sulfoniums were found highly stable in an artificial gastric juice (residual rate (%) of 1: 92.5 ± 6.1; 2: 91.4 ± 4.6; 3: 93.2 ± 6.2; 4: 96.5 ± 4.7, treated at 37 °C for 180 min). In addition, the sulfoniums were scarcely absorbed from the intestine in the experiment using *in situ* rat ligated intestinal loop model (residual rate (%) of 1: 97.6 ± 3.9; 2: 99.7 ± 6.0; 3: 94.5 ± 4.1; 4: 96.6 ± 3.7, treated for 120 min). These results indicate that sulfoniums (1–4) are promising leads for the new type of antidiabetes agents.

### 4.30. Lipidemic, Glycemic and Organ Protective Actions of Tea Seed Oil in Rats Fed with High Fat and High Carbohydrate Diet Thamolwan Suanarunsawat*, Warinna Pinthong, Wacharaporn Devakul Na Ayutthaya

The present study was conducted to investigate the lipidemic, glycemic and organ protective effects of tea seed oil (TSO) in rats fed with high fat and high carbohydrate (HFHC) diet. Three groups of male Wistar rats were used including normal control group, group fed with HFHC diet (cholesterol + lard + fructose) for three months, and HFHC group treated with TSO. At the end of the experiment, serum lipid profile, blood glucose, oral glucose tolerance test (OGTT), serum AST, ALT, LDH, CK-MB, creatinine and BUN were determined. Individual fatty acids containing in TSO was assayed by Gas chromatography. The results showed that oleic acid was the primary fatty acid in TSO (83.36%). HFHC diet increased serum lipid profile and atherogenic index (AI). The high serum levels of lipid profile and AI were decreased in HFHC rats treated with TSO. The elevation of area under the curve of OGTT was alleviated by TSO. TSO also normalized the high serum levels of AST, ALT, LDH, CK-MB, creatinine and BUN. It can be concluded that TSO was able to decrease the high serum levels of lipid profile, and high blood glucose level of OGTT in rats fed with HFHC, indicating its therapeutic potency to prevent atherosclerosis and hyperglycemia. It also protects liver, heart and kidney in HFHC. Oleic acid containing in TSO might be responsible for these activities.

### 4.31. Young Coconut Juice Phytoestrogens Alleviated Oxidative Stress and Dyslipidemia in Ovariectomized Mice Jitbanjong Tangpong*, Lanchakorn Chanudom, Monthakarn Thongsom

Postmenopausal women experience a markedly increase risk of lipid disorders and oxidative stress cause by decreasing of estrogen hormone. Hormone replacement therapy can lower that risk but can also increase the risk of cancer. Phytoestrogen from young coconut juice (YCJ) might be a safer alternative source of hormone replacement therapy. The purpose of this study aimed to investigate the effects of phytoestrogen in YCJ on hyperlipidemia and oxidative stress in ovariectomized mice. Mice were fed with YCJ at the concentration of phyotestrogen 100, 200, 400 and 800 pg/kgBW/day and synthetic estrogen 200 µg/kgBW/day, respectively. The results indicate that YCJ and synthetic estrogen significantly decreased blood glucose, triglyceride, cholesterol, LDL-cholesterol, lipid peroxide and increased blood HDL-cholesterol. Its anti-oxidative stress potential was therefore ameliorated glutathione peroxidase activity, superoxide dismutase activity, Glutathione levels and total antioxidant capacity. Moreover, YCJ treated groups showed no effect on gain body weight and significantly lower uterus weight compared to synthetic estrogen replacement therapy group. This study indicates the effects of estrogen-like hormone and antioxidant properties of YCJ which improved lipid metabolism and reduced oxidative stress in ovariectomized mice. Development of YCJ might be safety and benefit for the prevention and treatment of menopausal syndrome.

### 4.32. Mamao Pomace Alleviates Hypertension and Oxidative Stress in Nitric Oxide Deficient Rats Upa Kukongviriyapan*, Wanida Donpunha, Orachorn Boonla, Katmanee Senaphan, Poungrat Pakdeechote, Veerapol Kukongviriyapan, Patchareewan Pannangpetch, Jintana Sripui, Amporn Sae-Eaw

Hypertension is a risk factor for cardiovascular disease. Reactive oxygen species (ROS)-induced oxidative stress plays a major role in pathogenesis of hypertension. Mamao (*Antidesma* sp.) of the Stilaginaceae family is a well-known indigenous fruit in Thailand. Mamao juice and wine are the commercial products produced from mamao fruits. It has been demonstrated that mamao pomace (MP), a by-product generated from mamao fruits, contains large amount of antioxidant polyphenolic compounds. The present study was aimed to investigate the antihypertensive and antioxidative effects of MP in rats treated with *N*^ω^-nitro-l-Arginine Methyl Ester (l-NAME), an inhibitor of endothelial nitric oxide synthase (eNOS). Hypertension was induced in male Sprague-Dawley rats by giving l-NAME (50 mg/kg) in drinking water for 3 weeks. MP at a dose of 100 or 300 mg/kg was orally administered daily to animals simultaneously with l-NAME. Normal control rats receiving distilled water were served as normotensive animals. A marked increase in blood pressure, peripheral vascular resistance, and oxidative stress was found after l-NAME administration. MP in a dose-dependent manner significantly reduced blood pressure, increased hindlimb blood flow and decreased hindlimb vascular resistance in l-NAME treated rats (*p* < 0.05). A reduction in blood pressure was associated with increased eNOS protein expression and suppressed superoxide production. The present study provides the first evidence for the antihypertensive effect of MP and suggests that MP might be used as dietary supplement against hypertension and oxidative stress in nitric oxide deficient condition.

### 4.33. Anti-Obesity Activities of Ginseng Leaves (GS-LE2) Jun Young Kwak*, Se Chul Hong, Ji Hyun Yoo, Kun Hee Lee, Su Yeon Seol, Yong Sik Park, Jong Dae Park, Mi Kyung Pyo

In this study, the anti-obesity activity of GS-LE2 made from ginseng leaves was investigated in cell and animal model. GS-LE2 contains over 8.0% of ginsenoside Re. GS-LE2 inhibited insulin-induced 3T3-L1 adipocytes differention through oil red O-staining and α-glucosidase activity in a dose-dependent manner. The C57BL/6J mice induced by a high-fat diet for 6 weeks were randomly divided into 6 groups: a normal diet group (ND), a high-fat diet group (HFD), a high-fat diet with 10 mg/kg/day of xenical group (PC), and a high-fat diet with 100, 300, and 500 mg/kg/day of GS-LE2 group (GS-LE2-100, 300, 500) and then fed for 4 weeks. For the assessment of the anti-obesity effect, body weight, serum triglyceride, total cholesterol, leptin, and HDL-cholesterol were measured. After 4 weeks, the body weights in the HFD group significantly increased, while those of the GS-LE2-500 group decreased significantly (*p* < 0.05). The levels of serum triglyceride, and total cholesterol were significantly lower, but the level of HDL-cholesterol was significantly higher in GS-LE2-500 compared with the HFD. The serum leptin level was significantly lower in GS-LE2-500 than those of HFD. These results suggest that the GS-LE2 could be developed as a functional food material with anti-obesity effect.

### 4.34. Receptor Mechanisms of Thymoquinone-Induced Hypotension in Normotensive Rats Hussam A. Mizher*, Noriah Mohd Noor, Marwan Saad Azzubaidi

*Nigella sativa* seeds “black cumin” have been widely used in traditional medicine for diseases treatment including hypertension. Thymoquinone (TQ) is one of the major active constituents in its volatile oil. The objective of the current study was to confirm the blood pressure lowering effect of TQ, and to investigate its mechanism through muscarinic and β adrenergic receptors. Mean arterial blood pressure (MAP) was recorded using the non-invasive blood pressure tail cuff technique. Dose-response relationshipwas obtained after using 3 TQ doses (2.5, 5 and 10 mg/kg) intraperitoneally to 3 different groups (*n* = 5) of adult male Sprague Dawley rats under pentobarbital anesthesia. MAP was then measured for other 2 animal groups pretreated either with atropine (P-at) or propranolol (P-pro) followed by 10 mg/kg TQ. TQ produced a dose-dependent blood pressure lowering effect, where 2.5 mg/kg reduced MAP by 8 ± 1 mmHg, whereas 5 and 10 mg/kg of TQ treatment decreased MAP by 12 ± 3 and 29 ± 3 mmHg, respectively. TQ-induced MAP reduction was significantly less in P-at than non-pretreated group. Conversely, TQ-induced MAP reduction in P-pro did not demonstrate significant difference from the non-pretreated group. This study confirms the dose-related hypotensive effect of TQ and provides an evidence for the traditional use of *Nigella sativa* for treatment of hypertension. The mechanism of TQ-induced hypotensioninvolves at least in part activation of vascular muscarinic receptors, but not β-adrenergic receptors.

### 4.35. Inhaling Citronella Oil and Related Compounds and Their Effects on Rat’s Body Weight and Brown Adipose Tissue Sympathetic Nerve Activity Irmanida Batubara*, Irma H Suparto, Siti Sadiah, Ryunosuke Matsuoka, Tohru Mitsunaga

Citronella oil is one of the famous Indonesian essential oils with a distinctive aroma. As other essential oils, it is crucial to explore the effects of inhalation of this oil. Therefore, the aim of this research was to elucidate the effects of inhalation of citronella oil and its components isolated from *Cymbophogon nardus* (Sereh Wangi-Indonesian local name) on rat’s body weight, blood lipid profile, liver function, as well as the sympathetic nerve and temperature of brown adipose tissue. The inhalation of citronella oil, R-(+)-citronellal, and β-citronellol on *Sprague-Dawley* male adult rats fed with high fat diet (HFD) were performed for 5 weeks and compared to HFD rats without inhalation treatment. The results showed that inhalation of β-citronellol decreased the feed consumption. As a consequence, the weight gain decreased compared to the control group and significantly lowered the blood cholesterol level in the β-citronellol group. Liver function enzymes were not significantly different in all groups. The sympathetic nerve and temperature of brown adipose tissue of rats inhaled β-citronellol only small change. In conclusion, inhalation of citronella oil specifically β-citronellol decreased the body weight by decreasing the appetite without changes on liver enzymes concentration.

### 4.36. Anti-Obesity Effects of Red Ginseng Marc (GS-M7) Kun Hee Lee, Se Chul Hong, Ji Hyun Yoo, Su Yeon Seol, Yong Sik Park, Jong Dae Park and Mi Kyung Pyo*

Red ginseng marc (GS-M7) is the byproduct after extraction of red ginseng and is generally discarded as waste, even though it contains bioactive compounds such as acidic polysaccharides and lipid soluble components.In this study, the anti-obesity effect of GS-M7 was evaluatedin cell and animal model. GS-M7 reduced the adipocytes differentiation and lipid accumulationby oil red O-stainingand peroxisome proliferator-activated receptor-gamma (PPAR-r). For the assessment of the anti-obesity effect of GS-M7 in high fat diet mouse model, the mice were randomly divided into 6 groups: a normal diet group (ND), a high-fat diet group (HFD), a high-fat diet with 10 mg/kg/day of xenical group (PC), and a high-fat diet with 100, 300, and 500 mg/kg/day of GS-M7 group (GS-M7-100, 300, 500) and then fed for 4 weeks. High fat diet increased mouse weight, while GS-M7 reduced high fat diet-mediated increase of weight. In addition, the amounts of leptin, total cholesterol, and triglyceride were significantly reduced. However, HDL-cholesterol in GS-M7 treated group was higher than that of in HFD. In conclusion, it is thought that GS-M7 may have a potential for developing an anti-obesity agent.

### 4.37. Effects of Holarrhena antidysenterica as a Resistance Modifying Agent on Outer Membrane Permeability and Efflux Pumps against Acinetobacter baumannii Thanyaluck Siriyong*, Supayang Piyawan Voravuthikunchai, Sasitorn Chusri

*Acinetobacter baumannii* is the emerging multidrug resistant (MDR) and extensive drug resistant (XDR) pathogens responsible for nosocomial infections that are difficult to control and treat. The intrinsic resistance to broad-spectrum antibiotics due to the low permeability of the outer membrane as well as constitutive expression of efflux pumps, urgently needs to search new agents. Natural products as resistance modifying agents (RMAs) have been extensively reported. The mechanisms of *Holarrhena antidysenterica* and its main constituent, conessine as RMAs on bacterial outer membrane permeabilization was determined by using the fluorescence dye 1-*N*-phenylnaphthylamine (NPN) and the efflux pump inhibition was evaluated by the intracellular accumulation of ethidium bromide (EtBr) and pyronin Y against *A. baumannii*. The results showed that *Holarrhena antidysenterica* and conessine could not increase the uptake of NPN and EtBr accumulation on *A. baumannii* isolates, whereas they significantly increased the accumulation of pyronin Y (*p* < 0.05). Our results suggested that *Holarrhena antidysenterica* and conessine acted as efflux pump inhibitors to restore antibiotics activity against *A. baumannii*.

### 4.38. Immunomodulatory Effect of Rhodomyrtus tomentosa (Aiton) Hassk. Extract in Nile Tilapia (Oreochromis niloticus) against Streptococcus agalactiae Pinanong Na-Phatthalung*, Naraid Suanyuk, Supayang Piyawan Voravuthikunchai

Natural products from plant have been identified as an immunostimulant factor to control and prevent fish disease. The objective of this study was to evaluate the effect of commercial diet supplemented with *Rhodomyrtus tomentosa* (Ait.) Hassk. extract on immune response and disease resistance against *Streptococcus agalactiae* in Nile tilapia (*Oreochromis niloticus*). Fish were fed with commercial diet supplemented with the extract at the concentrations of 5, 50, and 500 mg/kg daily for 14 days. After oral administration of the extract, haematological (erythrocytes (RBC), leucocytes (WBC), haemoglobin, and haematocrit) and biochemical (serum protein, hemolytic complement, lysozyme, and reactive oxygen species acitivity) parameters were determined in blood and serum samples. The fish were challenged with *S. agalactiae* ST11 and mortality was recored for 14 days. The values of haematological and biochemical parameters were not significantly differ (*p* > 0.05) from the control values. With the exception of serum lysozyme activity, the treatment group at concentration of 50 and 500 mg/kg was significantly increased (*p* < 0.05) in lysozyme activity in fish serum. Furthermore, the experimental group fed with the diet supplemented with the extract at 500 mg/kg was reduced the mortality rate by 30% when compared to control group during the *S. agalactiae* challenge. The results indicated that *Rhodomyrtus tomentosa* supplementation may enhance the immunity in Nile tilapia against *S. agalactiae*.

### 4.39. Polymethoxyflavones Metabolism by Human Intestinal Bacterium Nayoung Kim*, Mihyang Kim, Jaehong Han

Polymethoxyflavones (PMFs) are a collection of flavones with methoxy functional groups exist in fruits and vegetables. Important biological activities of PMFs including antioxidant, anticancer, antiatherogenic, and antiplasmodial, were reported. Metabolism of dietary PMFs by intestinal bacteria was studied, because the PMF metabolites can have different biological activities from the substrate. A human intestinal bacterium metabolizing PMFs was isolated under anaerobic condition and identified as *Blautia producta* MRG-PMF-1 (KJ0788647) from 16S rRNA sequence analysis. Along with microbiological characterizations, reactivity of MRG-PMF-1 was studied. 5,7-dimethoxyflavone was demethylated to 5,7-dihydroxyflavone via 7-hydroxy-5-methoxyflavone, and 5,7,4ʹ-trimethoxyflavone was converted to 5,7,4ʹ-trihydroxyflavone through the sequential demethylation.

### 4.40. Induction of Programmed Cell Death by 1-Hydroxy-2-hydroxymethylanthraquinone from Coptosapelta flavescens on Giardia intestinalis Trophozoites Kruawan Hounkong*, Nongyao Sawangjaroen, Wipapan Kongyen, Vatcharin Rukachaisirikul

The Thai medicinal plants represent a rich source of potential antiparasitic compounds. *Coptosapelta flavescens*, commonly used to expel intestinal worm, was investigated for its anti-intestinal protozoa parasite. 1-hydroxy-2-hydroxymethylanthraquinone, purified by column chromatography from *Coptosapelta flavescens* was found to induce apoptosis-like programmed cell death on *Giardia intestinalis* trophozoite at 50% inhibitory concentration (IC_50_) of 0.42 µg/mL as performed by AnnexinV-FITC assay. This was comparable to the standard drug, metronidazole. The morphological change of *G. intestinalis* after exposure to 1-hydroxy-2-hydroxymethylanthraquinone and metronidazole were later observed by scanning electron microscopy. It was found that when treated *G. intestinalis* with 1-hydroxy-2-hydroxymethylanthraquinone and metronidazole at IC_50_ concentration the trophozoites presented with several abnormality such as membrane bleb and ventral disc damage. These results show that the 1-hydroxy-2-hydroxymethylanthraquinone from *C. flavescens* may be considered to be a source of new drugs for the therapy of intestinal protozoa diseases.

### 4.41. Induction of Apoptosis through Oxidative Stress-Related Pathways in MCF-7, Human Breast Cancer Cells, by Ethyl Acetate Extract of Dillenia suffruticosa Yin Sim Tor*, LatifahSaiful Yazan, JhiBiau Foo, Nurdin Armania, Yoke Kqueen Cheah, Rasedee Abdullah

Breast cancer is one of the most dreading types of cancer among women. Herbal medicine has becoming a potential source of treatment for breast cancer. Herbal plant *Dillenia suffruticosa* (Griff) Martelli under the family Dilleniaceae has been traditionally used to treat cancerous growth. In this study, the anticancer effect of ethyl acetate extract of *D. suffruticosa* (EADs) was examined on human breast adenocarcinoma cell line MCF-7 and the molecular pathway involved was elucidated. The cytotoxicity of EADs was determined by using MTT assay, mode of cell death by cell cycle analysis and apoptosis induction by Annexin-FITC/PI assay. Morphology changes in cells were observed under inverted light microscope. Involvement of selected genes in the oxidative stress-mediated signaling pathway was explored using multiplex gene expression analysis. The treatment of EADs caused cytotoxicity to MCF-7 cells in a dose- and time-dependent manner at 24, 48 and 72 h with IC_50_ of 76 ± 2.3, 58 ± 0.7 and 39 ± 3.6 µg/mL, respectively. Induction of apoptosis by EADs was dose- and time-dependent. EADs induced non-phase specific cell cycle arrest at different concentration and time point. The multiplex mRNA expression study indicated that EADs-induced apoptosis was accompanied by upregulation of the expression of *SOD1*, *SOD2*, *NF-κB*, *p53*, *p38 MAPK*, and *catalase*, but downregulation of *Akt1*. It is suggested that EADs induced apoptosis in MCF-7 cells by modulating numerous genes which are involved in oxidative stress pathway. Therefore, EADs has the potential to act as an effective intervention against breast cancer cells.

### 4.42. Cytotoxicity of Thymoquinone and Thymoquinone-Loaded Nanostructured Lipid Carriers on Breast Cancer Cell Lines (MDA-MB-231 and MCF-7) Latifah Saiful Yazan*, Yap Li Hua, Wan Nor Hafiza Wan Abd Ghani, Ng Wei Keat

Breast cancer is the leading cause of cancer death in women worldwide. Thymoquinone (TQ), one of the bioactives of the volatile oil of *Nigella sativa* (black seed), has been shown to exhibit anti-inflammatory, anti-oxidant and anti-tumour properties both *in vitro* and *in vivo*. Thymoquinone-loaded nanostructured lipid carriers (TQ-NLCs) were developed to improve the bioavailability and thus cytotoxicity of TQ. This study was conducted to compare the cytotoxic effects of TQ and TQ-NLC on breast cancer cell lines (MDA-MB-231 and MCF-7). Cytotoxicity of TQ and TQ-NLC towards the MDA-MB-231 and MCF-7 cells was determined by MTT assay. Cells were treated with TQ and TQ-NLC (3.125, 6.25, 12.5, 25, 50 and 100 µM) for 24, 48 and 72 h. Control (untreated cells) was included. The changes of cell morphology were observed under an inverted light microscope. Effects of TQ and TQ-NLC on the cell cycle were determined using a flow cytometer. Data showed that both TQ and TQ-NLC exhibited cytotoxicity and anti-proliferative activity towards MDA-MB-231 and MCF-7 cells in a dose-dependent manner. Both TQ and TQ-NLC were more cytotoxic towards MDA-MB-231 compared to MCF-7. The 50% inhibition of cell viability (IC50) of TQ in MDA-MB-231 cells for 24, 48 and 72 h was 3.83 ± 0.76, 3.93 ± 0.06 and 3.83 ± 0.06 μM, respectively. Meanwhile, the IC50 of TQ-NLC was 6.5 ± 0.5, 4.43 ± 0.12 and 4.46 ± 0.06 μM, respectively. The cytotoxic effect between TQ and TQ-NLC was found to be significant (*p* < 0.05). Cell shrinkage was noted following treatment with TQ and TQ-NLC. Cell cycle analysis showed an increase of apoptotic cell population in the treatment of both TQ and TQ-NLC compared to the control (*p* < 0.05) and induced non-phase specific cell cycle arrest at different exposure time. TQ was more cytotoxic than TQ-NLC. Both induced apoptosis and non-phase specific cell cycle arrest in MDA-MB-231 cells.

### 4.43. Cytotoxic Activity of Crude Extract from Piper cubeba L. against Breast Cancer Cell Lines Potchanapond Graidist*, Mananya Martla, Yaowapa Sukpondma

This study aimed to identify the chemical constituents from *Piper cubeba* L. and test the cytotoxic activities using MTT assay. Seeds were ground and soaked in methanol and dichloromethane. Methanolic crude extract was isolated by column chromatography. The fractionwas analyzed the structure by ^1^H-NMR. All fractions were tested cytotoxicity effect on normal and cancer cell lines. The most affective fraction was selected for DNA fragmentation assay to detect apoptotic activity. The methanolic crude extract showed higher cytotoxic activity against MDA-MB-468 and MCF-7 than dichloromethane crude extract. Methanolic crude extract was separated to 6 fractions namely A to F. Fraction C was highly active against MCF-7, MDA-MB-468 and MDA-MB-231 with IC_50_ value less than 4 µg/mL. Then, fraction C was isolated to 7 fractions namely CA to CG. The ^1^H-NMR profile showed that fraction CE is long hydrocarbon chains. Fraction CE demonstrated strong effect on cancer cell lines same as fraction C with IC_50_ value of 2.69 ± 0.09 µg/mL, 2.97 ± 0.15 µg/mL and 3.98 ± 0.12 µg/mL, respectively. In addition, fraction C and CE were tested with MCF-12A and showed IC_50_ value of 13.69 ± 2.36 and 2.91 ± 0.15, respectively. Finally, DNA fragmentation with a ladder pattern characteristic of apoptosis was observed in MDA-MB-468 and MCF-7 treated with fraction CE. Our results suggest that crude extracts (fraction CE) showed cytotoxic activity on breast cancer cell lines may be via apoptosis pathway.

### 4.44. Induction of Cell Cycle Arrest and Apoptosis in MCF-7 Cells by Dillenia Suffruticosa Root Extract via Reactive Oxygen Species Formation and Multiple Signaling Pathways Jhi Biau Foo*, Latifah Saiful Yazan, Yin Sim Tor, Nurdin Armania, Norsharina Ismail, Mustapha Umar Imam, Swee Keong Yeap, Yoke Kqueen Cheah, Rasedee Abdullah, Maznah Ismail

*Dillenia* *suffruticos* (Family: Dilleniaceae) root dichloromethane extract (DCM-DS) has been reported to exhibit strong cytotoxicity towards breast cancer cell lines. The present study was designed to investigate the cell cycle profile, mode of cell death and signaling pathways of DCM-DS-treated human MCF-7 breast cancer cells. Results showed that DCM-DS was cytotoxic to the MCF-7 cells in a time-and dose-dependent manner. The IC_50_ values of DCM-DS at 24, 48, and 72 h were 20.3 ± 2.8, 17.8 ± 1.5 and 15.5 ± 0.5 µg/mL, respectively. Cell cycle analysis revealed that DCM-DS induced G_0_/G_1_ and G_2_/M phase cell cycle arrest in MCF-7 cells at low concentration (12.5 and 25 µg/mL) and high concentration (50 µg/mL), respectively. Although Annexin-V/PI-flow cytometry analysis has confirmed that DCM-DS induced apoptosis in MCF-7 cells, the distinct characteristics of apoptosis such as membrane blebbing, chromatin condensation, nuclear fragmentation and formation of apoptotic bodies were not observed under microscope. DCM-DS induced formation of reactive oxygen species (ROS) in MCF-7 cells. Nevertheless, the co-treatment with antioxidants did not completely attenuate the cell death. The pro-apoptotic genes *JNK* and *P53* were up-regulated whereby anti-apoptotic genes *AKT1* and *ERK1/2* were down-regulated in a dose-dependent manner. For protein expression study, DCM-DS significantly up-regulated the expression of pro-apoptotic p53 and Bax but has no effect on the expression of anti-apoptotic Bcl2 in MCF-7 cells. As a conclusion, the activation of multiple signaling pathways by DCM-DS and ROS ultimately induced apoptosis in MCF-7 cells.

### 4.45. Purple Rice Extract Inhibits the Growth of Prostate Cancer Cell Lines by Suppression of Androgen Receptor Expression Teera Chewonarin*, Ratasak Summart, Chanarat Kiriya

The objective of this study was to investigate the effect of purple rice extract on the growth of androgen dependent human prostate cancer cell line (LNCaP). First, 3 varieties of purple rice (Nan, Phayao and Doisaket) and one of brown rice (RD.6) were extracted by methanol and dichloromethane. Known active compounds in all extracts were measured to find out the relation to biological activities. LNCaP was treated with both extracts of purple rice compared with RD.6 for 24 and 48 h. The result showed that at 50–200 µg/mL of methanol extract of all 3 varieties of purple rice slightly inhibited the growth of LNCaP while the extract of RD.6 had no effect. Next, the effect of purple rice extracts on expression of androgen receptor (AR) and prostate specific androgen (PSA) was determined by reverse transcription-PCR (RT-PCR). The results showed that expression of AR and PSA from only methanol extracts of purple rice treated cells at 6.25–50 µg/mL were decreased significantly while the dichloromethane extract showed less effect even if to 200 µg/mL. These results suggested that all methanol extract of purple rice had inhibitory effect on AR expression in mRNA level and downstream of AR responsive gene, PSA expression was also down regulated. Therefore, purple rice extract might be further developed as a food supplement for prostate cancer in aged people. However the molecular mechanism and active compounds in purple rice extract will be further investigated.

### 4.46. Neonothopanus nambi Speg., a New Source of Antibiotic and Anti-Inflammatory Agents Ampornrut Prapaiwong, Sukanlaya Leejae*, Teeratat Sudsai, Somporn Phonkrathok, Acharee Suksuwan, Prasan Tangyuenyongwatana, Krisana Kraisintu

*Neonothopanus nambi* Speg. is a poisonous basidiomycete luminescent mushroom. The information on antibacterial and anti-inflammatory activities of the fungus has not been established. Study on antibacterial and anti-inflammatory potencies of *N. nambi* extracts using *in vitro* model were assessed. The results indicated that the mycelia of the luminescent mushroom extracted with CHCl_3_, EtOAc, and culture filtrate extracted with EtOAc demonstrated excellent antibacterial activity against *Staphylococcus aureus* ATCC 25923 with minimum inhibitory concentration (MIC) and minimum bactericidal concentration (MBC) values ranged from 2–4 µg/mL, which is very closed to that of vancomycin (0.5–1 µg/mL). Moreover, hexane fraction of the collected mycelia exhibited obvious antibacterial potency against the pathogen with MIC and MBC values of 16 µg/mL. In contrast, all of the fractions were inactive against *Escherichia coli* ATCC 25922, a Gram-negative bacterium. For anti-inflammatory assay, CHCl_3_ fraction of *N. nambi* mycelia showed the most potent inhibitory effect on nitric oxide*-*released macrophage cells with an IC_50_ of 10.9 µg/mL followed by EtOAc and hexane fractions with IC_50_ values of 19.7 and 25.6 µg/mL, respectively. Both of the culture filtrate and the collected mycelia extracted with CHCl_3_ exhibited comparable effect to the positive controls, L-NA (NO synthase inhibitor, IC_50_ = 10.2 µg/mL), indomethacin (IC_50_ = 16.6 µg/mL), the standard used non-steroidal anti-inflammatory drugs. Purification, antibacterial mechanism of action, and anti-inflammatory activity of the extracts are still under investigations.

### 4.47. Ageratum conyzoides Leaf Extract Inhibit Inflammatory Response via Suppression of NF-B and MAPKs Pathway in LPS-Induced Macrophages Sawinee Seemakhan* and Klaokwan Srisook

*Ageratum conyzoides* (Asteraceae) has been widely used in traditional medicine in several countries for the treatment of skin diseases, ulcer wound, diarrhea, fever, pain and inflammation. Leaf extracts of *Ageratum conyzoides* have been shown anti-inflammatory activity in several *in vivo* models. However, the mechanism of its action has not been described yet. In this study, we determined the anti-inflammatory activity and the molecular mechanism of the ethanol extract of *Ageratum conyzoides* leaves (ACE) in lipopolysaccharide (LPS)-stimulated RAW264.7 macrophage model. ACE exhibited an inhibitory effect on inducible nitric oxide synthase (iNOS)-catalyzed nitric oxide (NO) and cyclooxygenase-2 (COX-2)-catalyzed prostaglandin E_2_ (PGE_2_) production with IC_50_ values of 23.4 and 18.5 µg/mL, respectively. ACE showed no significant cytotoxic effect determined by MTT assay. ACE attenuated the expression of iNOS and COX-2 at mRNA as well as protein levels in a concentration-dependent manner. Additionally, ACE suppressed the level of nuclear factor-κB (NF-κB) translocation and phosphorylation of p38 kinase, extracellular receptor kinase (ERK) and c-jun NH_2_ terminal kinase (JNK) of mitogen-activated protein kinases (MAPKs). These results indicate that ACE inhibits inflammatory response, at least in part, by inhibition of NO and PGE_2_ production through suppression of iNOS and COX-2 expression via a signaling pathway that involves NF-κB nuclear translocation and MAPKs phosphorylation. These findings provide the scientific evidence to justify the anti-inflammatory therapeutic use of *Ageratum conyzoides* leaves in traditional medicine.

### 4.48. Hepatoprotective Effect of Selected Egyptian Plant Extracts against Carbontetrachloride-Induced Hepatotoxicity in Experimental Models Hesham El-Askary*, Soheir El Zalabani, Miriam Yousif, Riham Salah El-Dine, Marwa Yousry Issa, Rehab Ragab Hegazy and Azza Hassan

Hepatoprotective efficiency of flax seeds-Lin (*Linum usitatissimum*), fenugreek seeds-Fen (*Trigonella foenum-graecum*) and rosemary leaves-Ros (*Rosmarinus officinalis*) were, *in-vitro* and *in-vivo*, assessed using Silymarin as positive control. Plant material were defatted with *n-*hexane (Lin1, Fen1 and Ros1) then extracted with 70% ethanol. Fenugreek and rosemary ethanol extracts (Fen2, Ros2) were partitioned between dichloromethane, ethyl acetate, *n*-butanol and water (Fen3-Fen6 and Ros3-Ros6). Lignan-rich fractions (Lin3 and Lin4) were, respectively, isolated from alkaline and acid hydrolysates of flax ethanol extract (Lin2). All samples (each in 10, 100, 1000 µg/mL), were tested, *in-vitro*, on human hepatoma cell-line (Huh7). Biochemical parameters (AST, ALT, SOD and GSH levels) were measured before and after CCl_4_-injury. *In-vitro* active samples (Lin3, Fen1, Fen5, Ros3, Ros6; each in 100, 200, 400 mg/kg) were, *in-vivo*, tested on CCl_4_ liver-injured rats, compared to ethanol extracts (Lin2, Fen2, Ros2; each in 200, 400, 800 mg/kg); biochemical (AST, ALT and total bilirubin) and histopathological changes were recorded. All samples, *in-vitro*, significantly reduced AST and ALT and restored GSH levels, meanwhile, highest increase in SOD level was observed for Lin3, Fen5 and Ros3. Although, all flax and fenugreek samples tested *in-vivo*, decreased AST (13.9%–59.3%), ALT (16.0%–36.7%) and total bilirubin (16.0%–48.6%), yet only low doses of rosemary samples were effective. Histological examination revealed the efficiency of flax and fenugreek over rosemary in preventing CCl_4_-induced inflammation, necrosis and vacuolation. In conclusion, fenugreek fractions (Fen1, 2, 5), flaxseed-lignan rich fraction (Lin3) and rosemary dichloromethane fraction (Ros3), exhibiting the highest hepatoprotective activities and could be suggested as suitable candidates for further standardization and processing.

### 4.49. Inhibitory on Xanthine Oxidase Activity of Apium graveolens L. Leaf Extracts Jaturon Thipwong*, Preyanuch Aiadsi, Benjamas Nupan and Kanokrat Saisa-ard

Uric acid is made from the breakdown of hypoxanthine and xanthine by enzymatic activity of xanthine oxidase (XO). The high level of uric acid in human body leads to formation of gout. Nowadays, the synthetic chemical drugs such as allopurinol is used to treat gout. However, allopurinol has many side effects. Accordingly, the alternative natural drugs of gout is introduced to be choice of treatment for reducing side effect of chemical drugs. In this study, we demonstrated *Apium graveolens* L. had a potent natural inhibitor to XO activity. The *Apium graveolens* L. was extracted by difference extraction solvents including water, methanol and 95% ethanol. The preliminary phytochemical screening revealed that all crude extracts contained steroids, tannins, saponins and alkaloids without terpenoids. At the concentration level was 100 µg/mL, all crude extract had the inhibitory activity more than 80%. IC_50_ value was calculated by using non-linear regression equation. The ethanolic crude extract was exhibited the highest activity with IC_50_ of 1.65 ± 0.46 µg/mL, followed by methanolic and water crude extract with IC_50_ of 6.91 ± 0.85 and 33.58 ± 3.65 µg/mL, respectively. A phamacokinetic study found that *Apium graveolens* L. might be a therapeutic use in traditional folk medicine against xanthine oxidase-related diseases, in particular, gout.

### 4.50. Rice Bran Protein Hydrolysates Improve Insulin Resistance and Adipokine Secretion in Rats with Metabolic Syndrome Kampeeporn Boonloh, Veerapol Kukongviriyapan, Bunkerd Kongyingyoes, Upa Kukongviriyapan, Supawan Tavornchinsombut and Patchareewan Pannangpetch*

A high carbohydrates or fat diet causes an insulin resistance (IR) and metabolic syndrome (MS). Recently, rice bran diet has been demonstrated to have anti-dyslipidemia, anti-hypertensive and anti-atherogenic properties in obese mice model. In the present study we investigated the effect of rice bran protein hydrolysates (RBP) on insulin, adiponectin and leptin secretions which play an important role in IR in high carbohydrate-high fat (HCHF) induced MS rats. Male Sprague-Dawley rats weighing 200–230 g were used. The normal control group was fed on chow diet, the MS group was fed on HCHF diet with 5% fructose drinking water for 12 weeks. After that, the MS group was divided into 3 subgroups which were orally administered with RBP 500 mg/kg, pioglitazone 10 mg/kg or distilled water for further 6 weeks. Then, fasting blood glucose (FBG) and serum lipid profiles, insulin, adiponectin and leptin were determined. RBP 500 mg/kg significantly reduced FBG, total cholesterol (TC), triglyceride (TG) and serum leptin as compared to the MS-control group (*p* < 0.05). Furthermore, we found that serum insulin and an indicator of IR, HOMA-IR score of MS rats receiving RBP were significantly lower than that of MS-control (*p* < 0.05). Interestingly, the levels of adiponectin were increased while the levels of leptin were decreased significantly in MS rats receiving RBP and pioglitazone as compared to MS-control group. In conclusion, these results indicate that RBP ameliorates insulin resistance and improves adipokines’ secretions in the MS. RBP could be further developed as novel food supplement for metabolic syndrome patients.

### 4.51. Peptides-Derived from Thai Rice Bran Improves Endothelial Function in 2K-1C Renovascular Hypertensive Rats Orachorn Boonla*, Phattharaphon Tuangpolkrung, Upa Kukongviriyapan, Poungrat Pakdeechote, Veerapol Kukongviriyapan, Patchareewan Pannangpetch and Supawan Thawornchinsombat

In recent years, a growing number of studies have been seeking for complementary medicine treatment from dietary nutrients for hypertensive patients. Rice bran protein hydrolysate extracted from rice has been reported to be a rich source of bioactive peptides. Based on a recent report in an *in vitro* study, it was found that peptides-derived from Thai rice bran (RBP) possess antioxidant and angiotensin-converting enzyme inhibitory activities. The present study aimed to investigate the vasorelaxant and antihypertensive effects of RBP in a rat model of two kidney-one clip (2K-1C) renovascular hypertension. Male Sprague-Dawley rats were induced 2K-1C hypertension by placing a silver clip (0.2 mm i.d.) around the left renal artery, whereas sham-operated rats were served as control animals. After renal artery clipping for 6 weeks, the aortic rings were isolated for testing the vasorelaxant effect of RBP on the presence and absence of endothelial cells. Regarding the antihypertensive testing, 2K-1C and sham-operated rats were intragastrically administered with RBP (250 or 500 mg/kg) or distilled water continuously for 6 weeks. It is apparent that RBP induced endothelium-dependent vasorelaxation. Administration of RBP to 2K-1C rats significantly reduced blood pressure and decreased peripheral vascular resistance (*p* < 0.05), suggesting that the reduction in blood pressure might be due to the vasodilating effect of RBP. A significant decrease in lipid peroxidation and increase in antioxidant glutathione were found in 2K-1C rats treated with RBP. Moreover, decreased eNOS expression in 2K-1C hypertensive rats was significantly improved after RBP administration. Overall findings of this study suggest that RBP possesses antihypertensive property which is mainly due to endothelium-dependent vasodilatory action.

### 4.52. Stimulation of Dermal Fibroblast Collagen Synthesis in Vitro by Saponin Enriched Extract from Soybeans Sarunya Laovitthayanggoon*, Siripen Jarikasem and Sarinthip Muensaen

Soybean (*Glycine ma*x L.) is a species of legume native to East Asia, widely grown for its edible bean which has numerous uses. It has been used as a source of human food of high quality protein and other nutrients for hundreds of years. Recently, soyasaponin, one of the significant bioactive constituents from soybeans, has received more attention to be used in skin care cosmetic products due to its relevant benifical properties including antioxidation, anti-inflammation and water retention. Previous works have also reported anti-wrinkle property of certain saponins of which effect on collagen synthesis is of interest. In this study, saponin enriched extract from soybeans prepared by use of Diaion HP-20 macroporous resin was investigated for *in vitro* stimulating effect on type 1 collagen synthesis. The human dermal fibroblasts cell line (ATCC CRL-1744) was treated with three different concentrations (50, 100, 200 µg/mL) of the extract for 24 h and then the amount of type 1 collagen was measured using ELISA test kit. The results revealed that the percentage of stimulating effect on collagen synthesis after saponin enriched extract treatment were 8.08 ± 1.23 and 21.12 ± 1.54 at concentrations 100 and 200 µg/mL, respectively while that of a positive control ascorbic acid at 50 µg/mL was 31.08 ± 0.28. The present study demonstrates the collagen synthesis stimulating potential of soysaponins and provides a possibility to develop as anti-skin aging agent in cosmetic products.

### 4.53. Mangiferin Content in Coffee Leaves from Ceará, Minas Gerais and Two Commercial Sources Ricardo Farias Almeida*, Cornelia M. Ullrich, Robert Wyn Owen and Maria Teresa Salles Trevisan

Mangiferin has high potential as a cancer chemopreventive agent but readily available sources are scarce. Therfore the concentration of mangiferin and its isomer were quantited in the *Coffea arabica* leaves in Brazil—Ceará (Guaramiranga), Minas Gerais (Bom Sucesso and Manhumirim)—and two available commercial sources. Mangiferin is a flavonoid described recently in the leaves of seven of the 23 species *Coffea* and in many other herbal species with many potential pharmacological properties. Some of its pharmacologic properties are: cytoprotective, anti-oxidant, antimutagenic, anti-inflammatory, antidiabetic, antitumor, antiviral, immunomodulatory, vascular modulatory, anticancer and anti-HIV activities, lipid peroxidation inhibitor, and neuroprotective effects. Mangiferin is a *C*-glycosylxanthone which is highly resistant to hydrolysis, and can be bioavailable intact. The amount of total mangiferin in methanol extracts of the Brazilian species was in the range 0.34–2.34 g/kg. In 90% of cases mangiferin accounted for 80% or over of total mangiferins. Total mangiferin content tended to be considerably higher in coffee leaves obtained from trees growing in plantation under natural full-sun conditions compared to other types of management such organic treatment. Additionally in one comparison with a species from Minas Gerais (Bom Sucesso), young leaves contained much higher concentration (271%; 2.34 *vs.* 0.63 g/kg) than old leaves. Infusions studies with powder leaves of a commercial Brazilians source show that release of mangiferin is temperature dependent and that release at 100 °C is virtually instantaneous and equivalent to that of prolonged methanol extraction. Consumption of coffee tea leaf brews, as a natural source of mangiferin, may contribute significantly to elevated intake of this potentially health promoting phenolic compound.

### 4.54. Development of Antioxidant Soluble Drinking Powder from Mamao (Antidesma ghaesembilla) Fruit Extract Pongtip Sithisarn* and Wandee Gritsanapan

*Antidesma ghaesembilla* or mamao is a plant in Euphorbiaceae family. This plant has reddish purple fruits which are edible and become more popular in the recent years as beverages and health products. This experiment was set up in order to investigate free radical scavenging activities of extracts from the fruits of mamao prepared by different methods of extraction and drying including decoction, fresh squeezing and maceration using 1,1-diphenyl-2-picrylhydrazyl (DPPH) assay. Folin-Ciocalteu and pH differential methods were also conducted to quantitatively analyze total phenolic and total anthocyanin contents of the extracts. Finally, extract from the most suitable extraction and drying methods was selected for development of health supplement product. Decoction and drying by evaporation on a water bath promoted extract of mamao ripe fruits with the strongest free radical scavenging activity (IC_50_ = 72.42 ± 3.52 µg/mL) with high amount of total phenolic and total anthocyanin contents of 1.22 ± 0.36 g gallic acid equivalent in 100 g extract (g%GAE) and 7.09 ± 0.24 g cyanidin-3-glucoside equivalent in 100 g extract (g%C-3-GE), respectively. This extract was developed as soluble drinking powder by wet granulation method and qualitatively controlled by evaluations of loss on drying, thin layer chromatographic (TLC) and infrared spectroscopic (IR) fingerprints. The obtained product is pinkish red powder that contained 3.08 ± 0.81 g%C-3-GE in 1 sachet (14 g) and exhibited free radical scavenging activity equivalent to 0.004 g of vitamin C. The information from this study could be used as a guideline for the developments of antioxidant products from mamao fruit extracts in the future.

### 4.55. Application of Microencapsulated Bifidobacterium longum with Eleutherine americana in Fresh Milk Tofu Atchara Phoem*, Supayang Voravuthikunchai

*Bifidobacterium longum* was microencapsulated by extrusion technique and added in fresh milk tofu. Microencapsulation of *B. longum* with *Eleutherine americana* extract, *E. americana* oligosaccharides extract, and commercial fructo-oligosaccharides was evaluated for their survival after sequential exposure to simulated gastric and intestinal juices and refrigeration storage. The initial number of *B. longum* cells were in the range of 9.30–9.54 log_10_CFU/g beads. The beads made by extrusion technique were kept at 4 °C for 0, 2, 4, and 6 days. Microencapsulated *B. longum* with the extract and oligosaccharides extract in fresh milk tofu survived better than non-encapsulated cells under adverse conditions. The highest survival of viable cells was resulted from microencapsulation with the oligosaccharides extract. The viability of *B. longum* microencapsulation with the oligosaccharides extract decreased from 9.24 to 8.23 log_10_CFU/g after sequential incubation in the simulated juices on day 6. The survival of microencapsulated *B. longum* with the oligosaccharides extract was 9.24 log_10_CFU/g after refrigeration storage on day 6. This work suggested that microencapsulated *B. longum* with *E. americana* could enhance functional properties of fresh milk tofu.

### 4.56. Preparation of Eleutherine americana-Alginate Complex Microcapsules and Application in Bifidobacterium longum Atchara Phoem*, Supayang Voravuthikunchai

Microencapsulation using extrusion and emulsion techniques was prepared for *Bifidobacterium longum* protection against sequential exposure to simulated gastric and intestinal juices. *Eleutherine americana* extract, *E. americana* oligosaccharides extract, and commercial fructo-oligosaccharides were used as co-encapsulating agents. The beads made by extrusion and emulsion techniques were stored at 4 °C for 2 and 4 weeks. Initially, the number of viable *B. longum* was in the range of 9.37–9.67 log_10_CFU/g beads. The number of viable cells reduced after refrigeration storage. The survival of encapsulated cells before and after sequential incubation in the acidic and enzymatic conditions was better than that of non-encapsulated cells at weeks 0, 2, and 4. The viability of co-encapsulated cells before and after sequential exposure to the simulated juices was higher than that of encapsulated cells at weeks 2 and 4. The highest survival of *B. longum* was resulted from microencapsulation with the oligosaccharides extract. The survival of *B. longum* microencapsulation with the oligosaccharides extract made by extrusion and emulsion techniques decreased from 9.15–8.17 and 8.17–7.27 log_10_CFU/g, respectively after sequential incubation in the simulated juices at week 4. However, none of non-encapsulated cells survived after exposure to the simulated juices. This work suggested that microencapsulated *B. longum* with *E. americana* offers effective delivery of probiotic to colon.

### 4.57. Enhanced Bioactive Compounds in White Radish Sprout Using Chitosan Apidet Rakpenthai*, Meike Burow, Cark Erik Olsen, Kankao Karnpakdee, Supaart Sirikantaramas

White radish (*Raphanussativus*) contains many health-promoting compounds including glucosinolates and flavonoids. We investigated the effect of chitosan treatment on the metabolic change in white radish sprouts. The seeds were germinated in deionized water containing different concentrations of chitosan (5, 10, 20, and 40 ppm). Eight-day old sprouts were harvested for metabolite analysis using LC-MS, HPLC, and total phenolic assay. Partial least squares discriminant analysis (PLS-DA) adopted to model the data from LC-MS revealed that chitosan at all concentrations used affected the metabolite profiles of treated samples. The UV chromatogram at 320 nm showed the increased amounts of most compounds detected, which is in agreement with the total phenolic assay. Glucoraphasatin (4-methylthio-3-butenyl glucosinolate), the major glucosinolate, content detected by HPLC was also increased by 40% in the sprouts treated with 10 ppm chitosan. These results suggest that the chitosan application could be exploited to enhance the bioactive metabolites in white radish sprouts.

## 5. Author Affiliations

- Ahmat, N., Faculty of Applied Sciences, Universiti Teknologi MARA, 40450 Shah Alam, Selangor Darul Ehsan, Malaysia
- Almeida, R.F., National Center for Tumor Diseases, Im Neuenheimer Feld, 581/German Cancer Research Center (DKFZ), Germany
- Amnuaikit, T., Faculty of Pharmaceutical Sciences, Prince of Songkla University, Thailand
- Arsianti, A., Faculty of Medicine, University of Indonesia, Indonesia
- Balamurugan, K., Department of Biotechnology, Science campus, Alagappa University, India
- Bao, F., Department of Microbiology and Immunology/The Institute for Tropical Medicine, Kunming Medical University, China
- Batubara, I., Faculty of Mathematics and Natural Sciences, Bogor Agricultural University, Indonesia
- Boonla, O., Faculty of Medicine, Khon Kaen University, Thailand
- Carroll, A.R., Griffith School of Environment, Griffith University, Australia
- Cha, B.-J., Graduate School of Biotechnology & Department of Oriental Medicinal Materials and Processing, Kyung Hee University, Republic of Korea
- Chewonarin, T., Faculty of Medicine and Lanna Rice Center, Chiangmai University, Thailand
- Chirinang, P., School of Food Technology, Institute of Agricultural Technology, Suranaree University of Technology, Thailand
- De Kimpe, N., Faculty of Bioscience Engineering, Ghent University, Belgium
- Djajadisastra, J., Faculty of Pharmacy, University of Indonesia, Indonesia
- El-Askary, H., Faculty of Pharmacy, Cairo University, Egypt and Faculty of Pharmaceutical Sciences and Pharmaceutical Industries, Future University, Egypt
- Foo, J.B., Institute of Bioscience, Universiti Putra Malaysia, Malaysia
- Götz, F., Interfacultary Institute for Microbiology and Infection Medicine Tübingen (IMIT), Microbial Genetics, University Tübingen, Germany
- Graidist, P., Faculty of Medicine, Prince of Songkla University, Thailand
- Hahm, K.B., CHA University of Medicine and Science CHA Medical Center, Seongnam and CHA Cancer Prevention Research Center, Korea
- Han, J., Metalloenzyme Research Group and Department of Systems Biotechnology, Chung-Ang University, Korea
- Hounkong, K., Faculty of Science and Natural Product Research Centre of Excellence, Prince of Songkla University, Thailand
- Howe, P.R.C., Clinical Nutrition Research Centre and School of Biomedical Sciences and Pharmacy, University of Newcastle, Australia
- Ismail, N.I., Faculty of Biosciences and Medical Engineering, Universiti Teknologi Malaysia, Malaysia
- Jain, A., Department of Pharmaceutical Sciences, and Sri Sathya Sai Institute of Pharmaceutical Science, India
- Jayasuriya, W.J.A.B.N., Department of Medical Education and Health Sciences, University of Sri Jayewardenepura, Sri Lanka
- Joycharat, N., Faculty of Traditional Thai Medicine and Natural Products Research Center of Excellence, Prince of Songkla University, Thailand
- Jung, J.-W., Graduate School of Biotechnology and Research Institute of Life Sciences & Resources, Kyung Hee University, Korea
- Kayser, O., Technical University Dortmund,Technical Biochemistry, Germany
- Kim, N., Metalloenzyme Research Group and Department of Systems Biotechnology, Chung-Ang University, Korea
- Klungsupya, P., Department of Pharmaceuticals and Natural Products (PNPD), Thailand Institute of Scientific and Technological Research (TISTR), Thailand
- Kukongviriyapan, U., Faculty of Medicine, Khon Kaen University, Thailand
- Kwak, J.Y., International Ginseng & Herb Research Institute, Chungnam, Korea
- Kwon, J.-H., Graduate School of Biotechnology and Institute of Life Sciences & Resources, Kyung Hee University, Korea
- Lai, H.S., Institute of Biological Sciences, Faculty of Science, University of Malaya, Malaysia
- Laovitthayanggoon, S., Pharmaceutical and Natural Products Department, Thailand Institute of Scientific and Technological Research, Thailand
- Li, N., Macau Institute for Applied Research in Medicine and Health, Macau University of Science and Technology, Macao
- Leejae, S., Faculty of Oriental Medicine, Rangsit University, Thailand
- Luesch, H., Department of Medicinal Chemistry and Center for Natural Products, Drug Discovery and Development (CNPD3), University of Florida, USA
- Mizher, H.A., Faculty of Pharmacy, International Islamic University Malaysia, Malaysia
- Morikawa, T., Pharmaceutical Research and Technology Institute, Kinki University, Japan
- Mustafa, M.R., Faculty of Medicine, University of Malaya, Malaysia
- Musthafa, K.S., Natural Product Research Center of Excellence, Prince of Songkla University, Thailand
- Natakankitkul, S., Faculty of Pharmacy, Chiang Mai University, Chiang Mai
- Naowaboot, J., Faculty of Medicine, Thammasat University, Thailand
- Na-Phatthalung, P., Faculty of Science, and Natural Product Research Center of Excellence Prince of Songkla University, Thailand
- Pandian, S.K., Department of Biotechnology, Alagappa University, India
- Pannangpetch, P., Faculty of Medicine, Khon Kaen University, Thailand
- Park, J.-H., Graduate School of Biotechnology and Research Institute of Life Sciences & Resources, Kyung Hee University, Korea
- Park, K.H., Division of Applied Life Science (BK21 plus), IALS, Gyeongsang National University, Korea
- Phoem, A., Faculty of Science and Technology, Songkhla Rajabhat University, Thailand
- Promsong, A., Faculty of Medicine, Prince of Songkla University, Thailand
- Pyo, M.K., International Ginseng & Herb Research Institute, Korea
- Rakpenthai, A., Faculty of Science, Chulalongkorn University, Thailand
- Rhee, Y., Department of Health, Nutrition, and Exercise Sciences, North Dakota State University, USA
- Rupasinghe, H.P.V., Faculty of Agriculture, Dalhosuie University, Truro, NS, Canada
- Safuan, S., School of Health Sciences, University Science Malaysia, Malaysia
- Seemakhan, S., Faculty of Science and Center of Excellence for Innovation in Chemistry, Burapha University, Thailand
- Senaphan, K., Faculty of Medicine, Khon Kaen University, Thailand
- Serm, L.G., Faculty of Science, University of Malaya, Malaysia
- Shanmugam, M.K., Yong Loo Lin School of Medicine, National University of Singapore, Singapore
- Siriyong, T., Faculty of Science and Natural Product Research Centre of Excellence, Prince of Songkla University, Thailand
- Sithisarn, P., Faculty of Pharmacy, Mahidol University, Thailand
- Somsak, V., Faculty of Medical Technology, Western University, Thailand
- Song, Y.H., Division of Applied Life Science (*BK21 plus*), *IALS*, Gyeongsang National University, Korea
- Suanarunsawat, T., Faculty of Science, Rangsit University, Thailand
- Sugimori, K., Faculty of Medicine, Toho University, Japan
- Suresh, T.S., Faculty of Medical Sciences, University of Sri Jayewardenepura, Sri Lanka
- Suttajit, M., School of Medical Sciences, University of Phayao, Thailand
- Suzuki, T., School of Pharmacy, Nihon University, Japan
- Tan, X., Division of Applied Life Science (BK21 plus), IALS, Gyeongsang National University, Korea
- Tanaka, K., Department of Traditional Medicine, Toho University School of Medicine, Japan
- Tangpong, J., School of Allied Health Sciences and Public Health, Walailak University, Thailand
- Thipwong, J., Faculty of Science and Technology, Suratthani Rajabhat University, Thailand
- Toida, T., Graduate School of Pharmaceutical Sciences, Chiba university, Japan
- Tongco, J.V., College of Forestry and Natural Resources, University of the Philippines Los Baños, College, Philippines
- Tor, Y.S., Laboratory of Molecular Biomedicine, Institute of Bioscience, Universiti Putra Malaysia, 43400 UPM, Serdang, Selangor, Malaysia
- Viljoen, A., Department of Pharmaceutical Sciences, Tshwane University of Technology, South Africa
- Werner, R.G., University of Tuebingen, Germany
- Wiart, C., School of Pharmacy, The University of Nottingham, Malaysia Campus, Malaysia
- Wibowo, A., Faculty of Applied Sciences, Universiti Teknologi MARA, Malaysia
- Widyawati, T., School of Pharmaceutical Sciences, Universiti Sains Malaysia, Malaysia and Medical Faculty, University of Sumatera, Indonesia
- Wiriyachitra, P., Asian Phytoceuticals Public Company Limited 84/3 Northern Region Industrial Estate Chiang Mai-Lampang Highway, Thailand
- Yazan, L.S., Faculty of Medicine and Health Sciences, Universiti Putra Malaysia
- Zjawiony, J.K., Department of Pharmacognosy and Research Institute of Pharmaceutical Sciences, University of Mississippi, USA

